# The ESCRT-III machinery participates in the production of extracellular vesicles and protein export during *Plasmodium falciparum* infection

**DOI:** 10.1371/journal.ppat.1009455

**Published:** 2021-04-02

**Authors:** Yunuen Avalos-Padilla, Vasil N. Georgiev, Elena Lantero, Silvia Pujals, René Verhoef, Livia N. Borgheti-Cardoso, Lorenzo Albertazzi, Rumiana Dimova, Xavier Fernàndez-Busquets

**Affiliations:** 1 Institute for Bioengineering of Catalonia (IBEC), The Barcelona Institute of Science and Technology (BIST), Barcelona, Spain; 2 Barcelona Institute for Global Health (ISGlobal, Hospital Clínic-Universitat de Barcelona), Barcelona, Spain; 3 Department of Theory and Bio-Systems, Max Planck Institute of Colloids and Interfaces, Science Park Golm, Potsdam, Germany; 4 Department of Electronics and Biomedical Engineering, Faculty of Physics, Universitat de Barcelona, Barcelona, Spain; 5 Computational Biology Group, Eindhoven University of Technology, Eindhoven, The Netherlands; 6 Department of Biomedical Engineering and the Institute for Complex Molecular Systems, Eindhoven University of Technology, Eindhoven, The Netherlands; Johns Hopkins University Bloomberg School of Public Health, UNITED STATES

## Abstract

Infection with *Plasmodium falciparum* enhances extracellular vesicle (EV) production in parasitized red blood cells (pRBCs), an important mechanism for parasite-to-parasite communication during the asexual intraerythrocytic life cycle. The endosomal sorting complex required for transport (ESCRT), and in particular the ESCRT-III sub-complex, participates in the formation of EVs in higher eukaryotes. However, RBCs have lost the majority of their organelles through the maturation process, including an important reduction in their vesicular network. Therefore, the mechanism of EV production in *P*. *falciparum-*infected RBCs remains to be elucidated. Here we demonstrate that *P*. *falciparum* possesses a functional ESCRT-III machinery activated by an alternative recruitment pathway involving the action of PfBro1 and PfVps32/PfVps60 proteins. Additionally, multivesicular body formation and membrane shedding, both reported mechanisms of EV production, were reconstituted in the membrane model of giant unilamellar vesicles using the purified recombinant proteins. Moreover, the presence of PfVps32, PfVps60 and PfBro1 in EVs purified from a pRBC culture was confirmed by super-resolution microscopy and dot blot assays. Finally, disruption of the *PfVps60* gene led to a reduction in the number of the produced EVs in the KO strain and affected the distribution of other ESCRT-III components. Overall, our results increase the knowledge on the underlying molecular mechanisms during malaria pathogenesis and demonstrate that ESCRT-III *P*. *falciparum* proteins participate in EV production.

## Author summary

Malaria is a disease caused by *Plasmodium* parasites that is still a leading cause of death in many low-income countries, and for which currently available therapeutic strategies are not succeeding in its control, let alone eradication. An interesting feature observed after *Plasmodium* invasion is the increase of extracellular vesicles (EVs) generated by parasitized red blood cells (pRBCs), which lack a vesicular trafficking that would explain EV production. Here, by combining different approaches, we demonstrated the participation of the endosomal sorting complex required for transport (ESCRT) machinery from *Plasmodium falciparum* in the production of EVs in pRBCs. Moreover, we were able to detect ESCRT-III proteins adjacent to the membrane of the host and in EVs purified from a pRBC culture, which shows the export of these proteins and their participation in EV production. Finally, the disruption of an ESCRT-III associated gene, *Pfvps60*, led to a significant reduction in the amount of EVs. Altogether, these results confirm ESCRT-III participation in EV production and provide novel information on the *P*. *falciparum* protein export mechanisms, which can be used for the development of new therapeutic strategies against malaria, based on the disruption of EV formation and trafficking.

## Introduction

*Plasmodium spp* is the parasite responsible for malaria, a disease that, despite the efforts done to control it, still represents a health problem worldwide particularly in low-income countries [1]. During *Plasmodium* infection, an elevated number of extracellular vesicles (EVs) from numerous cellular sources are circulating in the plasma [2], the amount of which correlates with the severity of the disease [2–5]. Despite of its high impact in the development of the pathology, the precise mechanism of EV formation in the infected red blood cells (RBCs) remains to be elucidated. One of the yet unsolved enigmas of malaria pathophysiology is how mature RBCs are able to release high amounts of EVs after *Plasmodium* infection, since they are biochemically simple compared to other eukaryotic cells and lack a normal vesicular network. It has been suggested that *Plasmodium* uses its own protein network to establish a vesicular trafficking for the export of an arsenal of virulence factors which contributes to the establishment of the parasite in the host cells [6].

In higher eukaryotes, EVs are generated and transported to their final destination by the endomembrane system [7]. Trafficking within the endomembrane system is crucial for the functional communication between different compartments in eukaryotic cells [8]. Depending on their origin and size, EVs can be classified into two major classes: exosomes and microvesicles. Exosomes refer to endosome-derived vesicles with a diameter typically of 30–50 nm that are generated following the fusion of multivesicular bodies (MVBs) with the plasma membrane. On the other hand, microvesicles are plasma membrane-derived vesicles which result from direct membrane shedding and exhibit a size from 100 nm up to 1 μm [9].

MVBs are shaped after the formation of intraluminal vesicles (ILVs) in early endosomes [10]. The genesis of ILVs relies on the sequential action of the endosomal sorting complex required for transport (ESCRT), which consists of four protein complexes termed ESCRT-0, ESCRT-I, ESCRT-II, ESCRT-III and a set of accessory proteins [11,12]. The best-described mechanism of ESCRT action begins with the recognition of mono-ubiquitinated proteins by ESCRT-0 [13], which then activates the recruitment of ESCRT-I [14] and ESCRT-II [15] that are responsible for membrane deformation into buds [16,17]. Finally, the polymerization of ESCRT-III begins with the binding of Vps20 to the invaginated membrane, which recruits the rest of the ESCRT-III members to the bud neck and the nascent vesicle is closed [16,18,19]. The membrane fission, protein dissociation and recycling of the machinery depends on the participation of the Vps4 AAA-ATPase [20]. Among all the sub-complexes, ESCRT-III (composed by Vps20, Snf7/Vps32, Vps24 and Vps2) and its accessory proteins (Vps4, Vta1, Vps60, Alix) are also involved in other important membrane-scission mechanisms, including virus budding, cytokinesis, nuclear envelope remodeling and exosome biogenesis among others (see review in [21]). All of these processes share the same topology where the nascent vesicle buds away from the cytosol, contrary to the topology observed in clathrin-coated vesicles [22].

The ESCRT machinery is highly conserved across the eukaryotic lineage; however, strictly intracellular protists, like *Plasmodium spp*, are devoid of ESCRT-0, -I and -II sub complexes [23]. In the case of *Plasmodium* and other organisms that lack the full ESCRT machinery, it is plausible that other proteins trigger ESCRT-III activation. In this regard, Alix, a Bro1-domain protein, binds directly to Vps32 and triggers the formation of ESCRT-III polymers, leading to ILVs formation in humans [24]. Whether a similar mechanism, alternative to the canonical ESCRT-III pathway, exists in *Plasmodium* remains to be determined.

Previous *in silico* assays showed that *Plasmodium falciparum*, the deadliest human malaria parasite species, possesses at least two putative proteins from the ESCRT-III complex: Vps2 and Vps32/Snf7 [23,25]. Additionally, the ATPase Vps4, an accessory protein of the ESCRT-III complex, was found in the cytoplasm of *P*. *falciparum* during the trophozoite blood stage [26]. Moreover, PfVps4 retained its function in MVB formation when transfected into *Toxoplasma gondii* and COS cells, thus strongly suggesting the existence of a functional ESCRT machinery in *P*. *falciparum* that mediates the production of MVBs [26].

Since *P*. *falciparum* lacks upstream ESCRT complexes, here we have investigated the presence of a putative Bro1-domain protein involved in an alternative ESCRT-III recruitment pathway. In addition, we have studied the participation of a minimal ESCRT-III machinery in EV biogenesis during *Plasmodium* infection. Overall, our findings provide an important insight into export mechanisms in *Plasmodium-*infected RBCs mediated by the parasite.

## Results

### *Plasmodium falciparum* possesses a Bro1 domain-containing protein

A previous *in silico* study in the *P*. *falciparum* genome revealed the presence of only six out of the 26 ESCRT-machinery proteins present in humans. The study showed that the genome of *P*. *falciparum* encodes four Snf7-domain containing proteins [23], a conserved feature in all ESCRT-III members [27]. Based on our *in silico* Basic Local Alignment Search Tool (BLAST) analysis, the four proteins were denoted as PfVps32, PfVps60, PfVps2 and PfVps46 (S1 Table).

The absence of ESCRT-I- and -II-associated genes and of a Vps20 homologue in the genome of *P*. *falciparum*, suggested the existence of an alternative recruitment pathway in the parasite. Hence, we explored the presence of a Bro1 domain-containing protein in *P*. *falciparum* that could bind directly to the Snf7 candidates and trigger the activation of the ESCRT-III system in this parasite, similarly to the process regulated by Alix in humans [24]. An *in silico* search of the *P*. *falciparum* genome (http://www.plasmodb.org) showed that the parasite has a unique Bro1-containing homologue termed PF3D7_1224200 (hereafter referred to as PfBro1) with a 3175 bp open reading frame and carrying 4 introns. The open reading frame of PfBro1 encodes an 819 amino acid protein with a predicted molecular mass of 98,714 Da. Our further assays revealed that the amino acid sequence of full-length PfBro1 had an identity of 21.8% with Alix, whereas the Bro1 domain in PfBro1 exhibited a 23.6% identity with its human homologue. Despite of this low amino acid conservation, we identified several conserved residues of the two charged polar clusters which, in several Bro1 homologues, stabilize the Bro1 domain [28]. These residues include R51, Y70 and E116 from the first cluster, and E187 and K246 from the second cluster (S1 Fig). Importantly, PfBro1 showed conservation of the residue I144 (S1 Fig), which has been demonstrated to directly participate in the binding of Vps32 in *Saccharomyces cerevisiae* [28]. We then performed additional tertiary structure prediction assays, revealing that the full-length PfBro1 has a hypothetical hydrophobic tail in its C-terminal region (S2 Fig), which makes it a good candidate for the recruitment of ESCRT-III components at the level of the membrane. Moreover, a pentameric *Plasmodium* export element (PEXEL) motif was found in the N-terminal sequence of PfBro1 (S1 Fig, double underline), which is present in many exported proteins in *P*. *falciparum* [29].

### PfBro1 and PfVps32 are exported to the cytoplasm of the erythrocyte

In order to continue our characterization of the ESCRT-III machinery in *P*. *falciparum*, we focused on resolving the putative role of three proteins: (1) PfVps32, the most abundant protein in the ESCRT machinery. (2) PfVps60, whose human homologue, CHMP5, is able to bind directly to Brox [30] (a Bro1-containing protein found in exosomes of human urine [31]), and redistribute it to membrane-enriched fractions [30]; and (3) PfBro1 as their potential recruiter and activator. Consequently, genes encoding the aforementioned proteins were chemically synthesized and cloned into the appropriate vector, to induce and purify the corresponding proteins. Coomassie blue-stained gels confirmed the integrity of the purified proteins (S3 Fig) which were used for the rest of the experiments.

Rabbit polyclonal antibodies against the purified recombinant proteins were generated and used to detect their presence in extracts obtained from *P*. *falciparum* cultures during the intraerythrocytic stage. In Western blot assays, the specific antibodies recognized PfVps32, PfVps60 and PfBro1 recombinant proteins from induced bacterial lysates, and *Plasmodium* native proteins in pRBC lysates 30 hours post invasion (hpi) (S3 Fig). Using parasite extracts, antibodies against PfVps32 and PfBro1 detected bands of the expected molecular weights (26 and 98 kDa, respectively). However, similarly to other Snf7-containing proteins [32,33], PfVps60 was detected with a higher molecular weight (46 kDa) than that predicted by the amino acid sequence (27 kDa) (S3 Fig). Nevertheless, bacterially expressed PfVps60 also migrated in an identical manner, suggesting that the highly charged nature of the proteins could influence their electrophoretic migration [32]. Preimmune serum used as a control did not reveal any band in *P*. *falciparum* extracts (S3 Fig).

**Fig 1:**
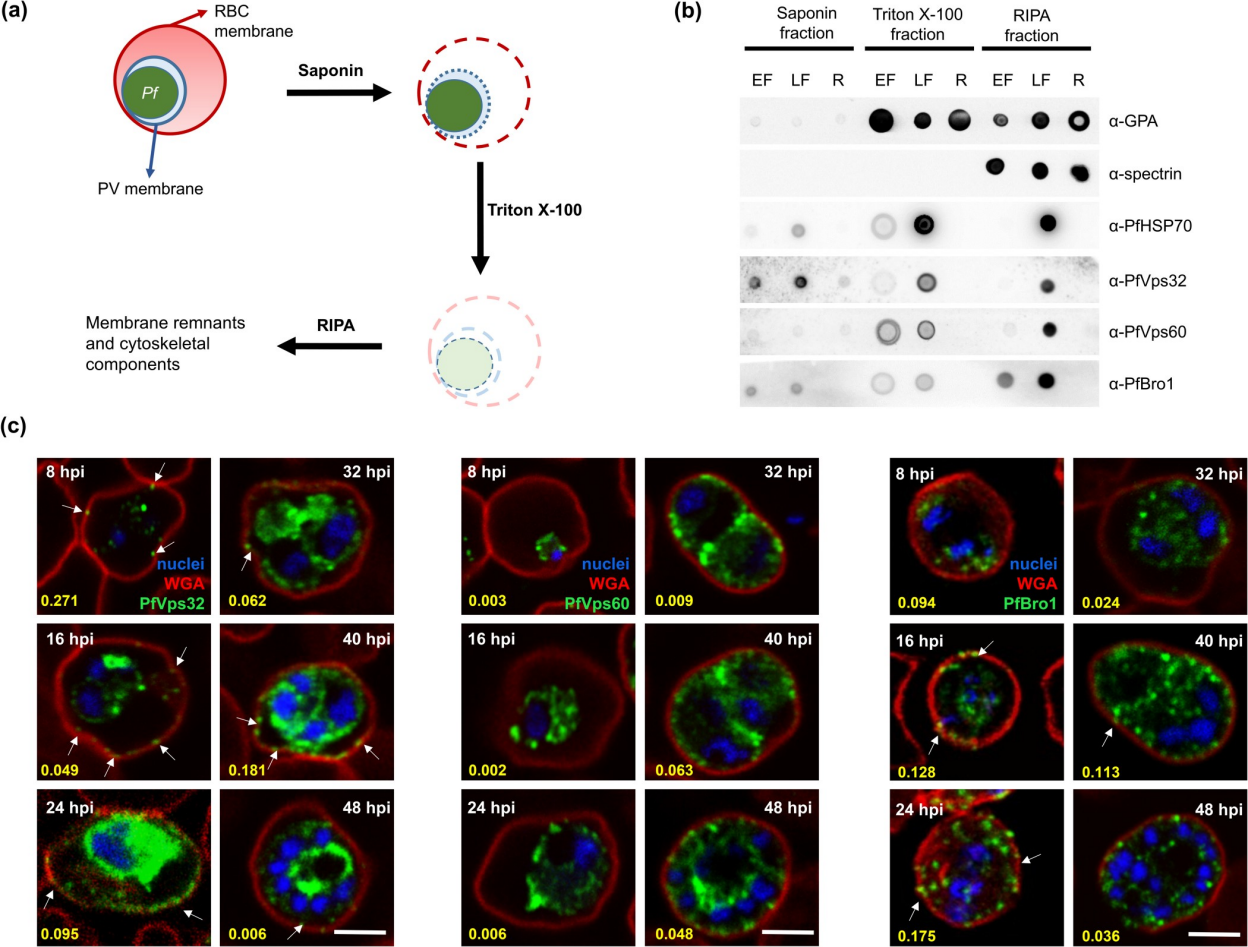
Expression and localization of PfVps32, PfVps60 and PfBro1 during the *P*. *falciparum* intraerythrocytic cycle. **(a)** Diagram illustrating the approach for the differential detergent fractionation protocol used for obtaining saponin, Triton X-100 and RIPA fractions. **(b)** Dot-blot assays performed with the different pRBCs extracts at early (8 hpi, EF) and late forms (40 hpi, LF) after RBC invasion using the different antibodies as indicated. As control, extracts obtained from non-infected RBCs (R) were also included. **(c)** Human erythrocytes were infected with *P*. *falciparum* and fixed at different hpi. PfVps32, PfVps60 or PfBro1 (green) were detected by indirect confocal immunofluorescence microscopy and WGA (red) was used to label the RBC plasma membrane. Cell nuclei were visualized with Hoechst 33342 (blue). Arrows show protein-labeled puncta adjacent to the membrane of the pRBCs. Numbers in yellow indicate Manders’ overlap coefficients used to evaluate co-localization between WGA and each protein tested. Scale bar: 5 μm.

To determine the localization of the studied proteins, parasites synchronized at early (8 hpi) or late stages (40 hpi) were lysed using a detergent fractionation approach. This technique allowed us to obtain three different fractions: (1) saponin fraction, containing RBC cytosolic proteins, (2) Triton X-100 fraction, enriched in proteins from membranes and *P*. *falciparum* organelles, and (3) radio-immunoprecipitation assay (RIPA) buffer fraction, where most cytoskeletal components are present (Fig 1A). To assess the purity of the isolated fractions, we used antibodies against glycophorin A (GPA), which is the major intrinsic membrane protein of the erythrocyte. As expected, GPA was absent from the saponin fraction but was detected in the Triton X-100 and RIPA fractions of both RBCs and pRBCs. On the other hand, antibodies against spectrin revealed the presence of this cytoskeletal protein only in the RIPA fraction of infected and non-infected RBCs.

PfVps32 and PfBro1 were present in the saponin extracts of both early and late stages after invasion, indicating their export to the RBC cytoplasm. In the RIPA buffer fraction, PfBro1 was found in early and late stages, while PfVps32 and PfVps60 were mainly present in late stages. Finally, all three proteins were found in the Triton X-100 extracts of both stages. As control, we used the *P*. *falciparum* heat shock protein 70 (PfHSP70), which was detected in all fractions of pRBCs except in the RIPA fraction at early stages. Neither ESCRT-III proteins nor the PfHSP70 control were present in protein fractions obtained from non-parasitized RBCs (Fig 1B). Western blot results indicated that PfVps32, PfVps60 and PfBro1 are expressed throughout the intraerythrocytic cycle (S4 Fig). Interestingly, antibodies against PfBro1 and PfVps60 detected more than one band in some fractions, which probably indicates a proteolytic processing of the proteins or their association to ligands possibly related with their function or degradation (S4 Fig). However, more experiments are necessary to prove this.

Immunofluorescence assays showed that PfVps32, PfVps60 and PfBro1 were localized in the cytoplasm of the parasite, inside the parasitophorous vacuole (PV) (Fig 1C). In the case of PfVps32 and PfBro1, puncta stained with their respective antibodies were observed in the cytoplasm of parasitized erythrocytes outside the PV (Fig 1C, arrows), some of them close to the RBC membrane. To examine whether these structures are exported to the parasitized RBC (pRBC) plasma membrane, lectins present in the RBC surface were labeled with wheat germ agglutinin (WGA) and Manders’ overlap coefficients were used to assess co-localization (Fig 1C, yellow numbers). There was no significant co-localization between WGA and the proteins, indicating that PfVps32- and PfBro1-labeled puncta localized adjacent to the surface lectin. The polyclonal antibodies raised against PfVps32, PfVps60 and PfBro1 did not recognize any structure in non-infected RBCs (S5A Fig). Neither the preimmune serum nor the secondary antibody controls displayed any signal in either RBC or pRBCs (S5B and S5C Fig).

### PfVps32, PfVps60 and PfBro1 are present in extracellular vesicles produced by pRBCs

**Fig 2:**
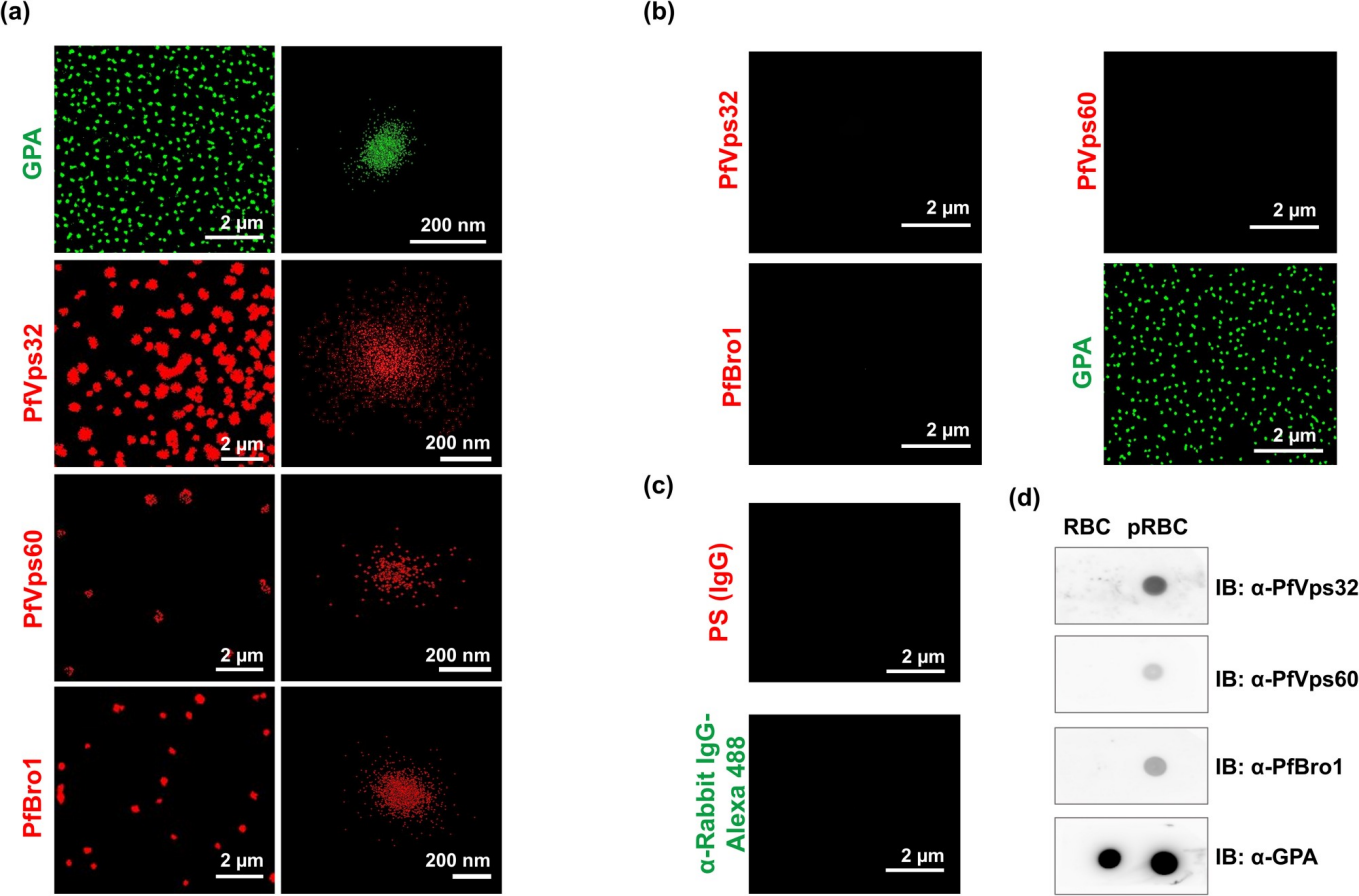
PfVps32, PfVps60 and PfBro1 proteins are present in EVs produced by pRBCs. **(a)** STORM detection by immunostaining of GPA, PfVps32, PfVps60 or PfBro1 in purified EVs derived from a 3% parasitemia, 3% hematocrit pRBC culture at 40 hpi. On the right column are shown images of individual EVs. **(b)** STORM detection as in (a) but using EVs derived from a 3% hematocrit RBC culture incubated for 40 h at 37°C. **(c)** Control of pRBC-derived EVs immunostained with preimmune serum (PS) or secondary antibodies against rabbit IgGs. **(d)** Dot blot assays performed in extracts of EVs derived from pRBCs (at 40 hpi) and RBCs (after 40 h of incubation at 37°C).

The results shown above suggested that ESCRT-III proteins could be involved in the export to the RBC cytoplasm of *Plasmodium*-derived proteins. To analyze whether ESCRT-III proteins were also present in EVs derived from pRBCs, we first evaluated by stochastic optical reconstruction microscopy (STORM) the presence of PfVps32, PfVps60 and PfBro1 in EVs derived from infected and non-infected RBCs. The higher resolution (~20 nm) of this technique compared to confocal microscopy (~ 0.5 μm), together with its high sensitivity, allowed us to precisely localize individual proteins in single EVs, whose size varies between 50 nm and 1 μm [9]. Parasite proteins were observed in EVs purified from a 3% parasitemia pRBC culture at 40 hpi (Fig 2A) and were absent in EVs from non-infected RBCs (Fig 2B), which confirmed our hypothesis and reflected ESCRT-III participation in *Plasmodium* EV biogenesis. To validate our approach, antibodies against GPA were used to detect EVs from RBC membrane origin (Fig 2A and 2B). Incubation of EVs derived from pRBCs with preimmune serum or secondary antibodies did not detect any signal (Fig 2C). STORM detection was confirmed by dot blot assays using proteins extracted from RBC- and pRBC-derived EVs. In this case, PfVps32, PfVps60 and PfBro1 were only detected in extracts from pRBC-EVs. As expected, control GPA was detected in EVs derived from both RBCs and pRBCs (Fig 2D).

### PfBro1 binds to membranes and recruits both PfVps32 and PfVps60 to trigger bud formation

So far, our results strongly suggested that there is a minimal ESCRT-III machinery participating in the formation of EVs in *P*. *falciparum*. Due to the fast binding and action of ESCRT-III proteins, it is difficult to assess their function in living cells. Other ESCRT-III mechanisms have been studied with the giant unilamellar vesicle (GUV) membrane model [33,34], which allows control of the lipid composition and visualization of the effects of ESCRT-III proteins on membranes by fluorescence microscopy.

To investigate whether *P*. *falciparum* ESCRT-III-related proteins were able to trigger membrane deformations, GUVs composed by palmitoyl-oleoyl-phosphatidylcholine (POPC) and palmitoyl-oleoyl-phosphatidylserine (POPS) (80:20) were generated to mimic the composition of the inner leaflet from the RBC plasma membrane [35]. We also included the fluorophore 1, 1’-dioctadecyl-3,-3,-3’,-3’-tetramethylindocarbocyanine perchlorate (DiI_C18_) to visualize membrane alterations (see Materials and Methods).

**Fig 3:**
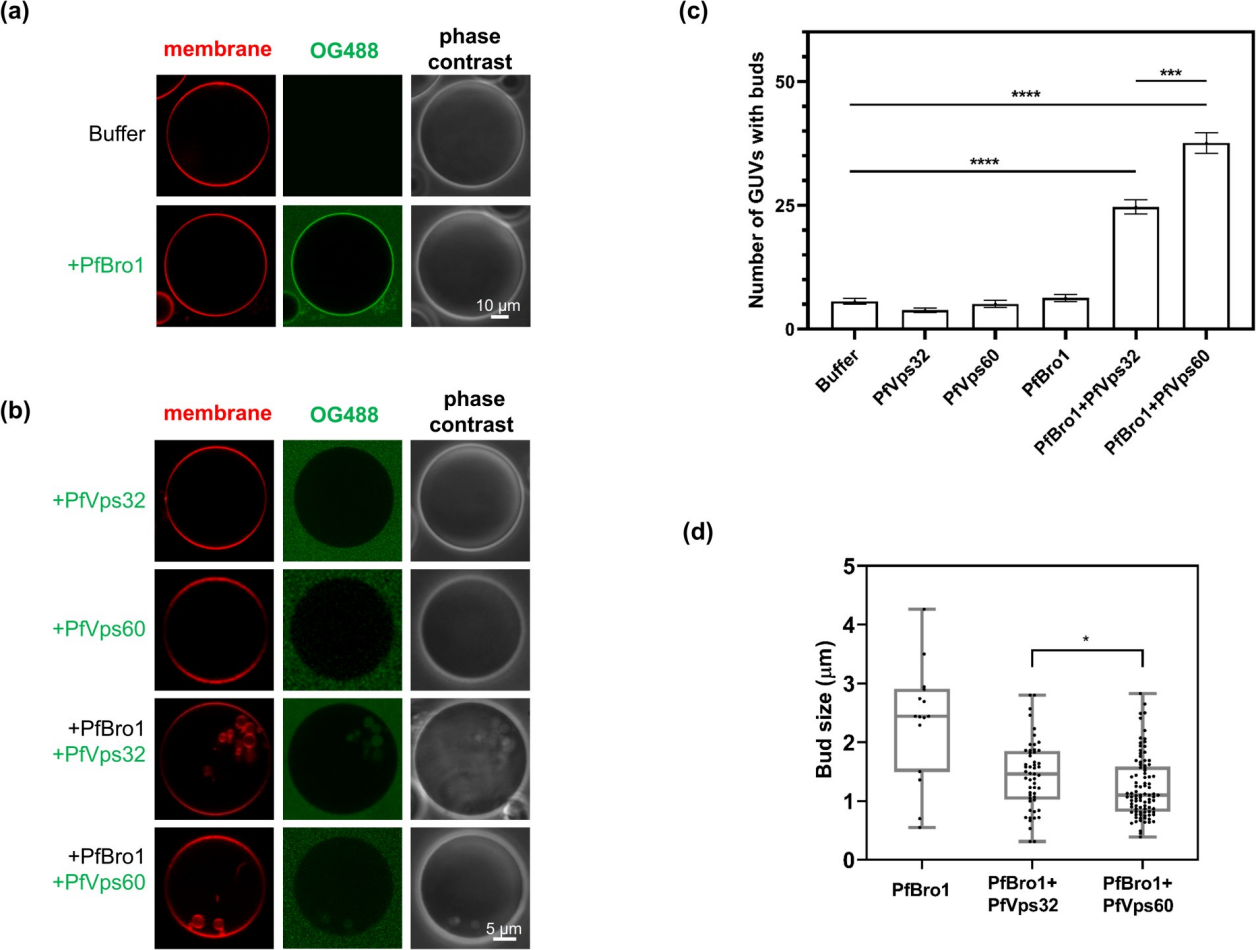
Intraluminal bud formation triggered by ESCRT-III *Plasmodium* proteins. GUVs composed by POPC:POPS (80:20), labeled with DiI_C18_ and diluted 1:2 in 2× protein buffer were incubated with **(a)** 600 nM PfBro1-OG488 with a 1:3 ratio of labeled and unlabeled protein, or **(b)** 1200 nM of either PfVps32 or PfVps60 labeled with OG488 (1:3 labeled:unlabeled) alone or in combination with 600 nM PfBro1 and visualized by fluorescence confocal microscopy. **(c)** Quantification of the number of GUVs with internal buds formed after protein addition. **(d)** Size of buds formed after the addition of the proteins indicated. Bars represent the mean and standard error of three independent experiments where 50 GUVs of each replicate were observed. *p* values were determined by Student’s t-test. *: *p* < 0.05, ***: *p* < 0.001, ****: *p* < 0.0001.

First, we tested the ability of PfBro1 to insert into lipid bilayers using its predicted hydrophobic sequence. When 600 nM of recombinant PfBro1 labeled with Oregon Green 488 (PfBro1-OG488) were incubated with POPC:POPS (80:20) GUVs diluted in an appropriate buffer, the protein inserted into GUV membranes with a homogenous distribution (Fig 3A). A truncated PfBro1 version lacking its hydrophobic domain (PfBro1t) failed to insert into GUV membranes (S6 Fig). Incubation in 150 mM NaCl, 25 mM tris-HCl, pH 7.4 (protein buffer) did not affect the GUV morphology (Fig 3A, top panel). After confirming PfBro1 binding to lipid bilayers, we investigated its role as a potential recruiter and activator of ESCRT-III proteins, in particular of Snf7-containg proteins. When POPC:POPS (80:20) GUVs were incubated with 600 nM of unlabeled PfBro1, followed by the addition of 1200 nM of either PfVps32 or PfVps60 labeled with Oregon green, the combination of both proteins induced the formation of intraluminal buds in the GUVs model (Fig 3B–3D). PfBro1+PfVps32-derived buds were significantly larger (1.43±0.51 μm) than those formed by PfBro1+PfVps60 (1.23±0.52 μm) (Fig 3d). Overall, these two bud types were smaller and more homogeneous in comparison to those where only PfBro1 was present (Fig 3D). Remarkably, the buds formed by PfBro1+PfVps60 exhibited a necklace-like arrangement and some tubular structures could be observed (see S1 Video). It is important to mention that the overall osmolarity of the mixture did not differ significantly after protein+buffer addition (~5 mOsmol/Kg change). As the incubation of GUVs with PfVps32 or PfVps60 only, either label-free of tagged with OG488, did not produce any detectable membrane changes (Fig 3B and 3C), we concluded that PfBro1 binds and activates both proteins.

### Putative activation of PfVps60 by PfBro1

It is well known that the activation of ESCRT-III subunits occurs after the displacement of the C-terminal domain that is blocking the binding site in the inhibited form of the protein [36]. The rearrangement of this domain has been documented to occur upon binding of activation factors such as Vps20, Vps32 or Bro1 [37]. To check whether this mechanism could be also operating in *P*. *falciparum*, we performed an *in silico* docking assay using the predicted tertiary structure of the Bro1 domain from PfBro1 and the full-length PfVps60 protein (see S1 Supporting Material and Methods for a full description of the methodology). This pair of proteins was selected because a higher number of GUVs with intraluminal buds was observed for this combination (Fig 3C). Upon binding to the PfBro1 domain, it was predicted that PfVps60 changed from a “closed” to an “open” conformation where the C-terminal domain modified its angle and allowed the exposure of the binding site (S7 Fig).

**Fig 4:**
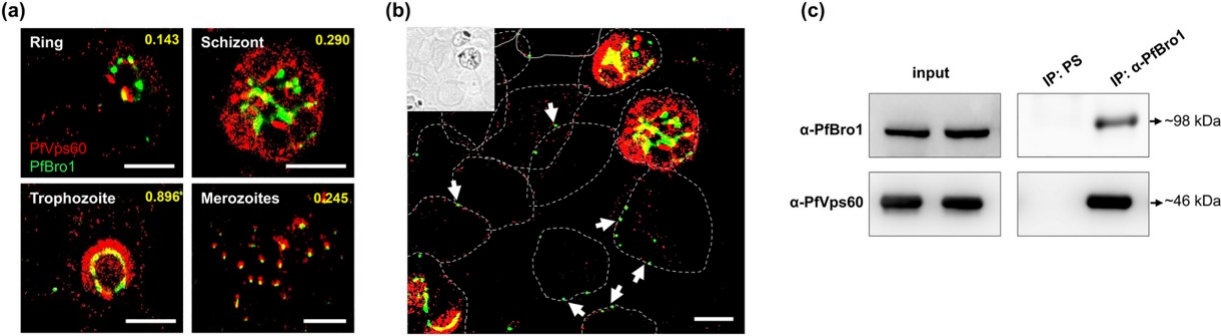
Interaction between PfVps60 and PfBro1. **(a)** STORM immunostaining detection of PfVps60 and PfBro1 in the blood stages of *P*. *falciparum*. **(b)** Field showing non-infected and infected RBCs. The inset contains a bright field low-resolution image to show the non-infected RBCs. Arrows pinpoint extracellular vesicles bound to non-infected RBCs, whose contours are indicated by dashed lines. Scale bars: 2 μm. **(c)** Immunoprecipitation of pRBC lysates at 30 hpi using anti-PfBro1 antibodies or preimmune serum (PS).

On the other hand, the colocalization of individual PfBro1 and PfVps60 molecules in pRBCs was further interrogated by STORM, taking advantage of the high sensitivity that this technique offers. Manders’ overlap coefficient demonstrated that PfBro1 and PfVps60 colocalized in the trophozoite stage of the intraerythrocytic cycle (Fig 4A). Interestingly, vesicles labeled with PfBro1 and PfVps60 were detected bound to the surface of non-infected RBCs (Fig 4B). Immunoprecipitation assays using anti-PfBro1 antibodies as bait confirmed the association of PfBro1 with PfVps60 in the trophozoite stage (Fig 4C). Altogether, these results indicated that PfBro1 is able to bind and activate PfVps60.

### PfBro1 and PfVps32 trigger bud formation by direct shedding from the plasma membrane

**Fig 5:**
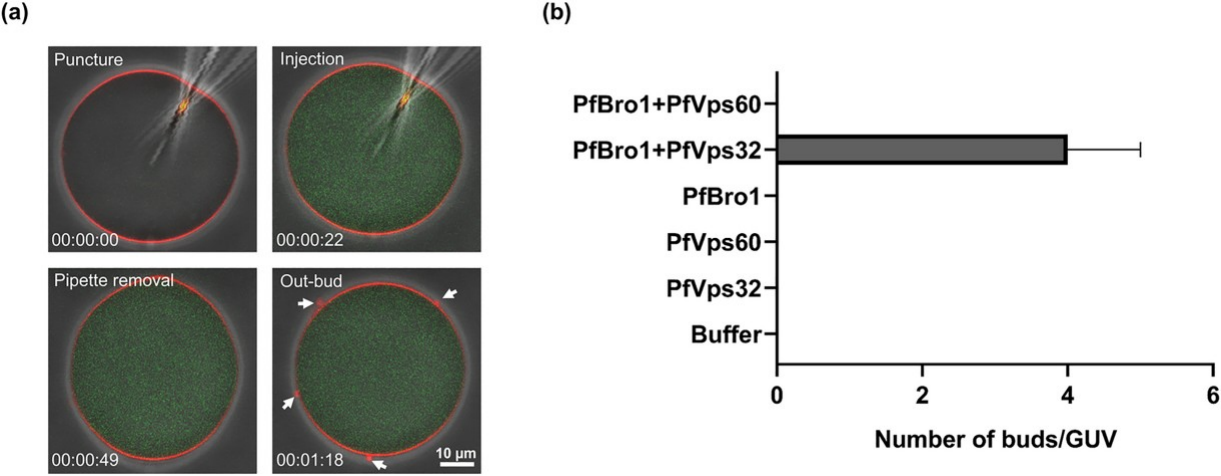
Injection of ESCRT-III *Plasmodium* proteins in GUVs and outward budding. **(a)** Panels show injection of a mixture of PfBro1 and PfVps32 (1:2) together with PEG-FITC in GUVs composed by POPC:POPS:DSPE-biotin (79:20:1) and labeled with DiI_C18_ (0.1 mol%). Four main events are presented: puncture, injection, pipette removal and generation of outward buds (arrows). **(b)** Graphical representation of bud number per GUV in the tested conditions for the injection approach. Bars represent the mean and standard error of the number of out-buds in four independent injected GUVs.

Using the purified recombinant proteins from *P*. *falciparum* and the GUV model we were able to reconstitute one of the two EV biogenesis pathways described in higher eukaryotic cells (MVB biogenesis; see review in [38]). However, the mechanism of microvesicle formation by direct shedding from the plasma membrane could not be reconstituted using this approach, and a microinjection strategy was designed for its study. For this, the biotinylated lipid 1,2-distearoyl-sn-glycero-3-phosphoethanolamine-N-[biotinyl(polyethylene glycol)-2000] (DSPE-PEG-biotin) was included in the lipid mixture to form GUVs containing protein buffer in their lumen (see Materials and Methods). GUVs were harvested and immobilized on an avidin-coated surface to allow their manipulation for injection. It is important to mention that prior to injection, a z-stack acquisition was performed in the confocal microscope to verify that GUVs lacked alterations in the membrane and that the contact area with the coverslip was not excessively large, which could compromise the assay (see example of a selected GUV in S8 Fig). As the labeling of proteins can compromise their activity, we used free polyethylene glycol fluorescein isothiocyanate (PEG-FITC) dye (0.03 mg/ml in protein buffer) to visualize the injection process. The incorporation of this control dye did not produce any detectable alterations in GUVs (see Figs 5 and S9, S2 Video). Upon injection of either PfBro1, PfVps32 or PfVps60 alone, or a combination of PfBro1 and PfVps60, no significant changes were observed in the membrane of the injected GUVs (Fig 5B). On the other hand, when PfBro1 and PfVps32 were injected together, the formation of extracellular buds was visualized in all the injected GUVs (Fig 5 and S3 Video). Contrary to the experiments observed in the previous approach (Fig 3), these buds appeared as single bodies with a homogeneous average size of 0.88 ± 0.076 μm, and remained attached to the mother vesicle moving along its surface (S3 Video).

### Disruption of PfVps60 causes a defect in EV production in *P*. *falciparum*

**Fig 6:**
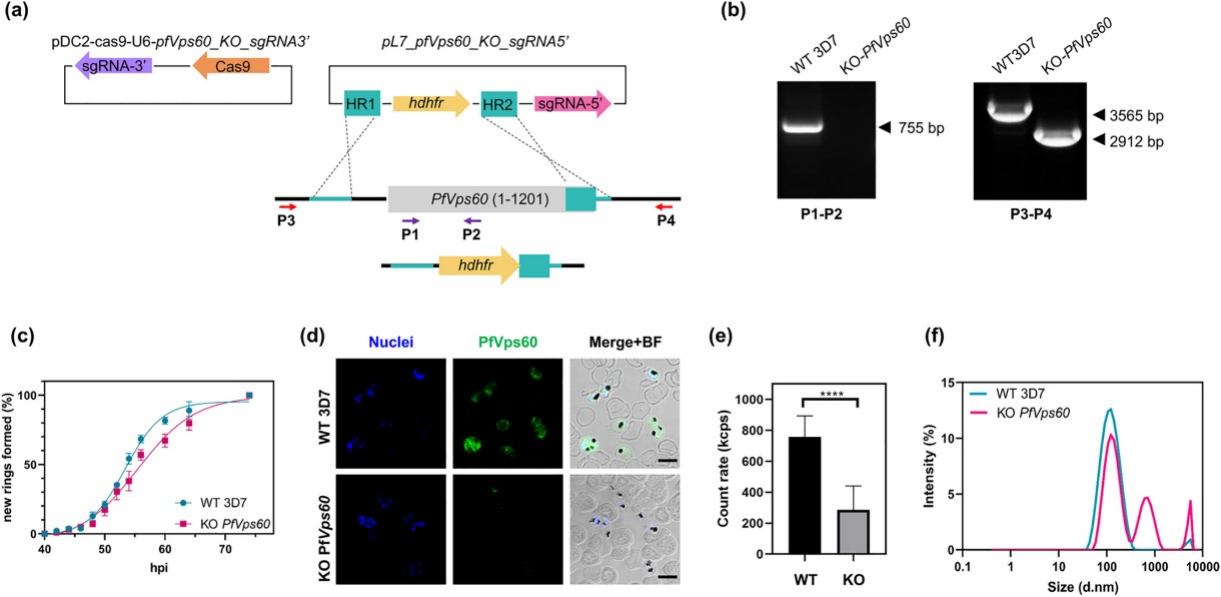
Generation and validation of *PfVps60* KO parasites. **(a)** Scheme of the strategy followed to generate the transgenic lines using the CRISPR/Cas9 system. Arrows indicate the position of primers used for diagnostic PCR. **(b)** Diagnostic PCR confirmation of the integration of the pL7-*PfVps60*_KO_sgRNA3’ plasmid at the *PfVps60* locus. Legends at the bottom of each panel indicate the primer pair used for each PCR reaction. Genomic DNA from the WT 3D7 line or the *PfVps60* KO transgenic line was used. The expected size of the bands is indicated on the right side of each panel. **(c)** Asexual blood cycle duration in the *PfVps60* KO line compared with its parental 3D7 line. Percentages indicate the proportion of rings observed relative to the total number of rings at the end of the assay. Data was fitted to a sigmoidal curve with variable slope to extract the intraerythrocytic developmental cycle. **(d)** Human erythrocytes infected with *P*. *falciparum* were fixed and PfVps60 (green) was detected by indirect immunofluorescence microscopy. Cell nuclei were visualized with Hoechst 33342 (blue). Fields were merged with bright field (BF) to assess localization. Scale bar: 5 μm. **(e)** Derived count rate of purified EVs from three independent replicates expressed in kilo counts per second (kcps). **(f)** Representative results of the size distribution of EVs derived from the WT 3D7 or the KO *PfVps60* strains determined using a Zetasizer Nano. Each symbol shows the mean of three different replicates, bars show the SE. ****: *p* < 0.0001.

Next, we evaluated the effect of ESCRT-III machinery inactivation on EV production by *P*. *falciparum*. While we failed at obtaining a stable strain for the KO of *PfVps32* and *PfBro1* (probably due to their essential role in the life cycle of the parasite), we succeeded in the establishment of a *PfVps60* KO strain by CRISPR/Cas9 gene edition (Fig 6A). Gene silencing and DNA integration were confirmed by diagnostic PCR as shown in Fig 6B. The relative fitness of the generated KO line was evaluated by a growth curve, which showed a slower progression in the KO line compared to its parental line (intraerythrocytic developmental cycle of 52.38 vs. 55.41 h, respectively; Fig 6C). The suppression of PfVps60 was confirmed by immunofluorescence assays, which indicated the absence of the protein (Fig 6D). In order to study the effects of the *PfVps60* gene disruption on EV production, EVs derived from WT and KO strains were purified in parallel and following the same protocol (see Materials and Methods). The resulting EV fractions were concentrated and resuspended to the same volume, and the amount of EV particles was determined. The number of EVs was significantly reduced in KO parasites in comparison to the parental 3D7 line (Fig 6E). Moreover, the measurements of particle size showed that the distribution of EVs differed between the KO and WT strains. In the WT, there were two main populations, one with a diameter of 40–300 nm and the other between 3–5 μm. In the KO, three populations with different diameters were revealed: 60–300 nm, 300–1,000 nm and 4,000–5,000 nm (Fig 6F).

**Fig 7:**
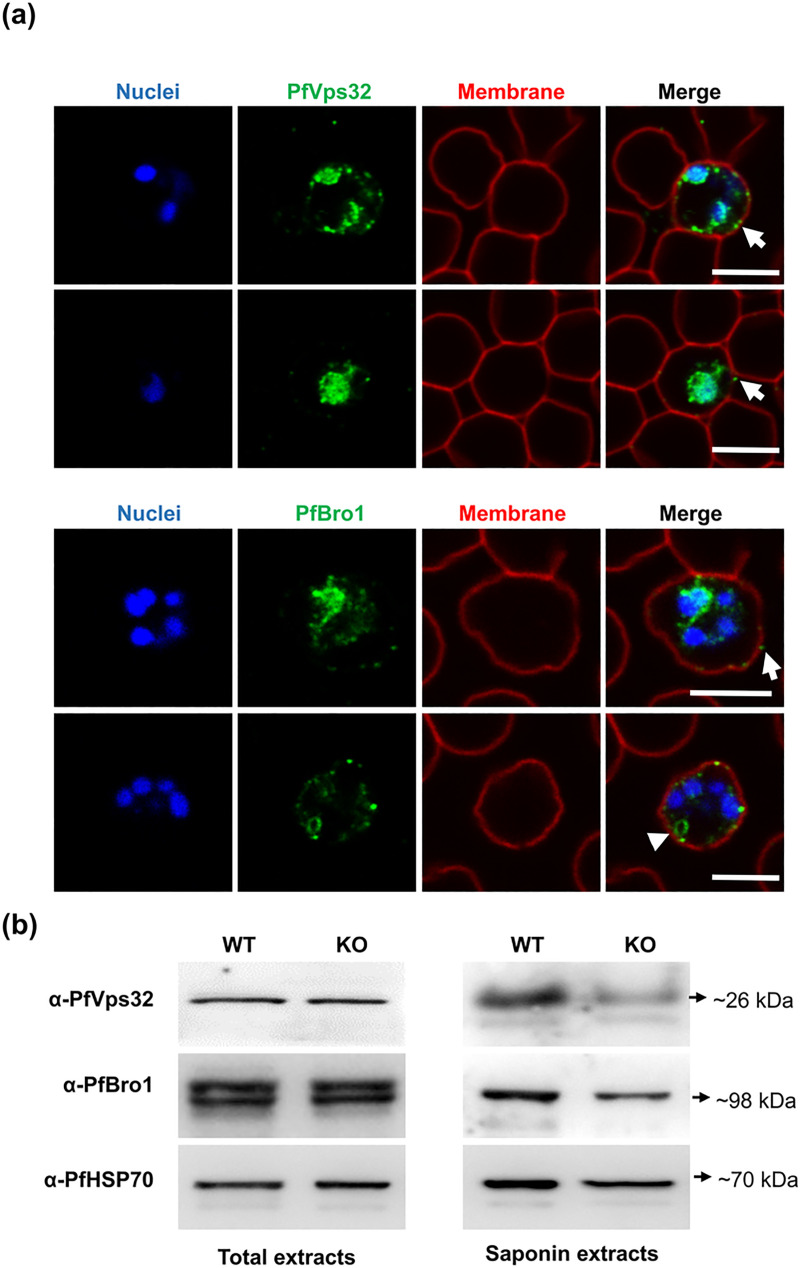
Effect on PfVps32 and PfBro1 by PfVps60 disruption. **(a)** Representative images of human erythrocytes infected with a *P*. *falciparum* KO-*PfVps60* strain and fixed with 4% PFA to detect PfVps32 and PfBro1 proteins (green) and WGA (red) by indirect immunofluorescence. Cell nuclei were visualized with Hoechst 33342 (blue). Arrows show protein-labeled puncta adjacent to the membrane of the pRBCs. The arrowhead shows a vesicle-like structure labeled with PfBro1. For each protein are shown two microscope fields corresponding to the KO strain. Scale bars: 10 μm. **(b)** Western blot assays using total protein extracts or the saponin fraction obtained from pRBCs infected with either the WT 3D7 or the KO-*PfVps60* strain to detect proteins indicated on the left side of the panels.

To assess whether the observed EV reduction was only due to the effect of PfVps60 absence or to a generalized disruption of the ESCRT machinery, we evaluated the distribution of PfVps32 and PfBro1 in the KO line. As shown in Fig 7A, both proteins appeared clustered around the periphery of the parasite, and in some cases large vesicular-like structures stained with PfBro1 were observed (Fig 7A, arrowhead). However, some PfVps32- and PfBro1-labeled puncta were observed adjacent to the RBC plasma membrane suggesting their export to the RBC cytoplasmic space. To confirm this, Western blot assays performed in total protein extracts 40 hpi revealed that the total amount of PfVps32 and PfBro1 remained unaltered in the KO strain (Fig 7B). However, we could observe a partial drop in the level of both proteins present in the saponin fraction, which confirmed that the proteins were still exported outside the PV but in lower amounts (Fig 7B).

## Discussion

Throughout its intraerythrocytic life cycle, *Plasmodium* resides within a PV where it needs to overcome the scarcity of nutrients and the absence of an exploitable cell machinery in the mature RBC. As a result, *Plasmodium* has evolved specialized and complex trafficking pathways, which allow the export of virulence-related proteins to the RBC cytoplasm or to the extracellular space [39]. Most of the exported proteins contain an N-terminal PEXEL motif and are carried outside the PV through a protein translocon inserted in the PV membrane [40]. However, other routes allow the transport of non-PEXEL proteins [41]. Among them, the secretory vesicle pathway that has been poorly investigated despite its participation in the transport of crucial proteins for the pathophysiology of the disease and in the transfer of drug resistance genes [42,43].

*Plasmodium*-infected erythrocytes increase the release of EVs, which participate in different pathogenic mechanisms (see review in [44]). Some proteins related with EV biogenesis have been elucidated, such as PfPTP2, which is involved in the intercellular communication between *P*. *falciparum* infected cells and that has been identified in vesicles in close contact with parasite-derived membranous structures present in the RBC cytoplasm, called Maurer’s clefts [43]. In higher eukaryotes, the ESCRT-III machinery is involved in both types of EV generation: exosome release and microvesicle budding [45,46]. However, the mechanisms underlying the release of EVs in *Plasmodium*-infected cells are far from being understood. The *P*. *falciparum* genome lacks genes encoding for ESCRT-III activating factors, such as Vps20 and ESCRT-II members [27]. Therefore, we hypothesize that there are alternative pathways for ESCRT-III activation in *P*. *falciparum* and most likely in other intracellular protists such as *T*. *gondii* and *Cryptosporidium parvum*, which also lack the aforementioned genes [23]. In *S*. *cerevisiae* and humans, Bro1 homologues are able to bind directly to the Snf7 domain of Vps32 and CHMP5 (Vps60 homologue) and activate ESCRT-III polymerization on the membranes [24,30,47]. In the present study, we show that *P*. *falciparum* possesses a Bro1 domain-containing protein, PfBro1, capable of activating two Snf7-domain containing proteins, PfVps32 and PfVps60, to trigger the formation of buds in the model of GUVs. These proteins are expressed throughout the intraerythrocytic cycle and are localized in the cytoplasm of the parasite inside the PV. However, PfVps32 and PfBro1 are also exported to the cytoplasm of the RBC, where they can participate in the biogenesis of microvesicles through plasma membrane shedding as discussed below. The identified PfBro1 has a hydrophobic sequence in its C-terminal region that allows its insertion into GUV lipid bilayers, thus making it a good candidate for ESCRT-III recruitment at the membrane. Moreover, we have also identified a conserved PEXEL motif in its N-terminal region (RNLKE) which could be involved in facilitating the export of PfBro1 from the PV to the RBC cytoplasm as already described for other PEXEL-containing proteins [48], however, more experiments are required to confirm this. On the contrary, PfVps32 and PfVps60 lack a PEXEL motif, which suggest that their export is carried out through a different mechanism.

**Fig 8:**
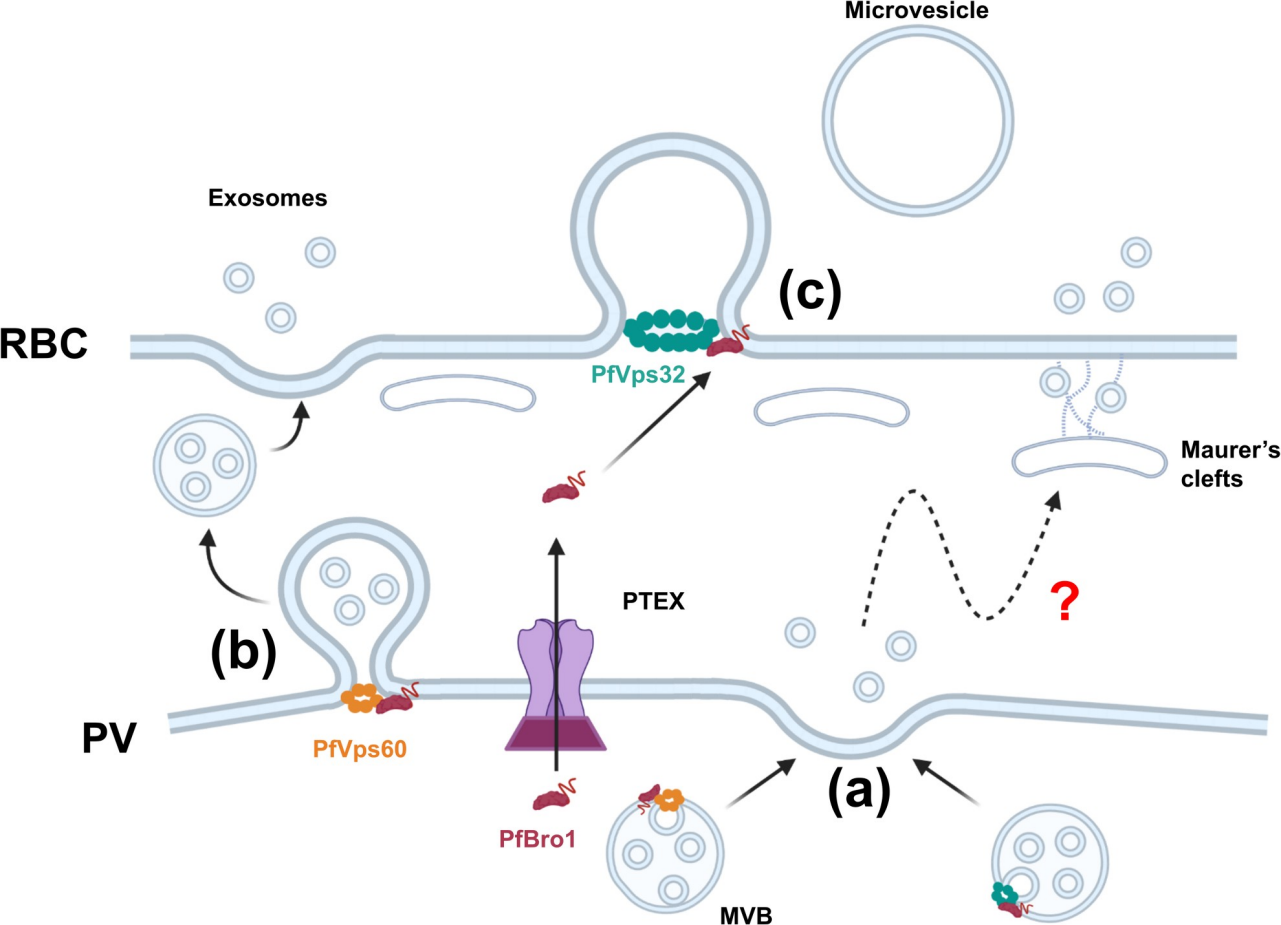
Proposed model for ESCRT-III-mediated EV biogenesis in *Plasmodium*. **(a)** PfVps32 and PfVps60 are recruited by PfBro1 and trigger the formation of intraluminal vesicles (ILVs) to generate MVBs. These MVBs fuse with the parasitophorous vacuole (PV) membrane and release the ILVs into the host cytoplasm through which they can be transported via the exo-membranous trafficking system present in pRBCs to be eventually released to the extracellular space. **(b)** MVBs are formed directly in the PV membrane after the insertion of PfBro1 which recruits PfVps60, generating MVBs that are released to the host cytoplasm. These MVBs eventually fuse with the RBC plasma membrane to release exosomes to the extracellular space. **(c)** PfBro1 can also be exported from the PV to the host cell through the PTEX. Once in the host cytoplasm, PfBro1 can insert into the RBC plasma membrane and recruit PfVps32 (which is exported by an as yet unknown mechanism) to initiate budding and trigger microvesicle formation. Created with BioRender.com.

The study of ESCRT-III interactions in living cells is problematic as the association between the different molecular components occurs in a fast manner and the protein complexes are difficult to obtain. Therefore, *in silico* docking assays were performed and the results showed that PfVps60 can shift from a closed (inactive) to an open (active) conformation upon PfBro1 interaction. This association was confirmed by STORM imaging and immunoprecipitation assays. Furthermore, we proved that PfBro1 is able to recruit both PfVps32 and PfVps60 to the GUV membrane and activate them, leading to the formation of buds, as occurs in the same model with other ESCRT-III homologues [34,49]. The purified proteins were used to recreate the two mechanisms of EV production (MVB generation and membrane shedding) in GUVs that mimic the composition of the inner leaflet of the erythrocyte plasma membrane. Interestingly, while bud generation is triggered by both PfVps32 and PfVps60 in a similar manner when the proteins are added outside the GUV, in the injection approach, we observed that bud formation was triggered only by the co-injection of PfBro1 and PfVps32. This discrepancy might be due to the requirement for a higher concentration of PfVps60 to drive membrane deformation, which cannot be achieved in the GUV femtoinjection approach. The presence of PfVps60 in the PV lumen would therefore suggest that the protein is present in sufficiently high amounts to allow its participation in MVBs formation inside the PV (Fig 8A) and in the budding of MVBs from the PV membrane (Fig 8B), both processes leading to exosome export. In GUV injection assays, PfVps32 was able to trigger bud formation at a fixed PfBro1 concentration, which reflects the efficiency of this protein to polymerize and deform membranes at low concentration. This scenario would not require that large amounts of PfVps32 are exported to the RBC plasma membrane for it to participate in the budding of microvesicles (Fig 8C).

We also observed that when the addition of PfVps32 or PfVps60 led to bud formation, the nascent vesicles varied in size depending on the protein used, with PfVps32 leading to significantly bigger buds in comparison to PfVps60. In higher eukaryotes there are several factors governing the size of ESCRT-III-derived buds, including Vps4 disassembly action [50,51], size of cargo [34] and membrane tension [49]. In the case of *P*. *falciparum*, whether the size of EVs is regulated by other ESCRT proteins encoded in its genome or by membrane biophysical properties, remains to be explored in the future. On the other hand, as the newly formed buds remained in close contact with the mother vesicle, we hypothesize that more factors are needed to release the nascent vesicle from the membrane.

The detection of PfBro1, PfVps32 and PfVps60 in purified EVs from a pRBC culture suggests that the ESCRT-III machinery participates in their biogenesis. Furthermore, silencing of the *PfVps60* gene resulted in the reduction of the number of EVs produced during the first 40 hpi, which indicates the participation of PfVps60 in EV biogenesis during *Plasmodium* infection. Although our initial aim was to inactivate the whole ESCRT-III complex, we could not obtain stable KO lines for PfVps32 and PfBro1 proteins, probably due to their involvement in essential processes from the parasite, most likely in cytokinesis as occurs in other eukaryotes [50,52]. The increase in the microvesicle population (<300 nm) observed in the KO strain could reflect that either other EV-production mechanisms in *P*. *falciparum* are enhanced or an alteration in the export mechanisms is produced upon *PfVps60* silencing. On the other hand, the subcellular distribution of PfVps32 and PfBro1 changed significantly with the disruption of the *PfVps60* gene. Both proteins were clustered around the periphery of the parasite, although their export to the RBC cytoplasm was conserved but in a reduced degree. It has been demonstrated that the depletion of *Vps* genes in other organisms resulted in mislocalization of MVB cargoes to the limiting membrane of the vacuole or to large aberrant structures called class E compartments [53,54]. STORM imaging allowed us to detect EVs labeled with PfBro1 and PfVps60 at the surface of non-infected erythrocytes, thus suggesting their involvement in a potential pathogenic priming mechanism that could facilitate invasion of targeted cells.

In conclusion, we propose that the mechanism of ESCRT-III-mediated EV biogenesis in *Plasmodium* starts at the PV lumen. In here, PfVps60 and PfBro1 might trigger the formation of MVBs where proteins destined for export are sorted in the intraluminal vesicles (ILVs). The release of ILVs can possibly occur by two different mechanisms: (1) MVBs formed in the PV lumen fuse with the PV membrane (PVM) and release the ILV into the host cytoplasm where they can be transported to the RBC plasma membrane via the exo-membranous trafficking system present in pRBCs (Fig 8A, see review in [55]), or (2) MVBs are formed directly in the PVM after the insertion of PfBro1, generating MVBs that are released to the host cytoplasm which latter fuse with the RBC plasma membrane to release exosomes to the extracellular space (Fig 8B). On the other hand, PfBro1 can also be exported from the PV to the host cell through the *Plasmodium* translocon of exported proteins (PTEX). Once in the host cytoplasm, PfBro1 can insert into the RBC plasma membrane and recruit PfVps32 (which is exported by an as yet unknown mechanism) to initiate budding and trigger microvesicle formation (Fig 8C).

Altogether, these results strongly suggest that both types of EV formation are being carried out in *Plasmodium*-infected RBCs, thus supporting previous observations [43,56]. Our results improve the mechanistic understanding of protein export in *P*. *falciparum*, and suggest that the proteins studied here represent a potential target for new therapeutic strategies to control malaria.

## Materials and methods

More information is available in the Supporting Materials and Methods.

### *P*. *falciparum* culture and synchronization

Unless otherwise indicated, reagents were purchased from Sigma-Aldrich (St. Louis, MO, USA). Asexual stages of *P*. *falciparum* 3D7 were propagated in group B human erythrocytes at 3% hematocrit using Roswell Park Memorial Institute (RPMI) medium supplemented with 0.5% (w/v) Albumax II (Life Technology, Auckland, New Zealand) and 2 mM L-glutamine. Parasites were maintained at 37°C under an atmosphere of 5% O_2_, 5% CO_2_ and 90% N_2_. For all experiments, the parasitemia of the culture was kept between 3 and 5%.

For tight synchronization, the parasite culture was initially synchronized in the ring stage with a 5% sorbitol lysis [57] followed by a second 5% sorbitol lysis after 36 h. Then, 36 h after the second sorbitol, parasites were synchronized in the schizont stages by treatment in 70% Percoll (GE Healthcare, Uppsala, Sweden) density centrifugation at 1,070 × g for 10 min. Finally, after the third synchronization a final 5% sorbitol lysis was done, yielding parasites tightly synchronized at 8 hpi.

### STORM

A 5% parasitemia RBC culture was prepared for super-resolution microscopy as described in [58]. Briefly, a μ-Slide 8 well chamber slide (Ibidi) was coated for 20 min at 37°C with 50 mg/ml concanavalin A. Then, wells were rinsed with pre-warmed phosphate buffered saline (PBS) before parasite seeding. Infected RBCs were washed twice with PBS and deposited into the wells. Cells were incubated for 10 min at 37°C and unbound RBCs were washed away with three PBS rinses. Seeded RBCs were fixed with pre-warmed 4% paraformaldehyde at 37°C for 20 min. After this time, cells were washed with PBS and then, incubated with polyclonal antibodies anti-PfVps60-Alexa Fluor 488 (1:500) and anti-PfBro1-Alexa Fluor 647 (1:1000). Finally, nuclei were counterstained with Hoechst 33342 (2 μg/ml).

Before STORM acquisition, the buffer was exchanged to OxEA buffer (3% v/v oxyrase, 100 μM DL-lactate, 100 mM β-mercaptoethylamine, dissolved in 1× PBS, pH 8.4) [59]. STORM images were acquired using a Nikon N-STORM system configured for total internal reflection fluorescence imaging. Excitation inclination was tuned to adjust focus and to maximize the signal-to-noise ratio. Alexa Fluor 647 and 488 were excited, respectively, illuminating the sample with 647 nm and 488 nm laser lines built into the microscope. Fluorescence was collected by means of a Nikon 100×, 1.4 NA oil immersion objective and passed through a quad-band-pass dichroic filter (97335 Nikon). 20,000 frames at 50 Hz were acquired for each channel. Images were recorded onto a 256×256 pixel region (pixel size 160 nm) of a CMOS camera. STORM images were analyzed with the STORM module of the NIS element Nikon software.

### Reconstitution of ESCRT-III in GUVs

GUVs containing POPC, POPS, and the fluorophore DiI_C18_ (Invitrogen, CA, USA) (80:20:0.1) were prepared in 600 mM sucrose as described previously [33]. Briefly, the lipid mix was spread on tin oxide-coated glass slides, and electro-swelling was performed for 1 h at room temperature (RT) at 1.2 V, and 10 Hz. All lipids were obtained from Avanti Polar Lipids (Alabaster, IL, USA).

For PfBro1 binding assays, GUVs were harvested and diluted 1:1 with 2× protein buffer (50 mM tris-HCl, 300 mM NaCl, pH 7.4). After 10 min of equilibration, GUVs were incubated with 600 nM of either PfBro1- or PfBro1t-OG488. For PfVps recruitment, equilibrated GUVs were incubated with 600 nM of PfBro1 and 1200 nM of either PfVps32 or PfVps60 with at least 10 min of incubation at RT between the additions of each protein. Images were acquired with a Leica TCS SP5 confocal microscope (Mannheim, Germany). DiI_C18_ was excited with a 561 nm laser and OG488 with a 488 nm line of an Argon laser. To avoid crosstalk between the different fluorescence signals, a sequential scanning was performed. All experiments shown in the same figure were done with the same GUV batch for comparability. Each experiment was repeated on at least three separate occasions with different batches of GUVs.

### Femtoliter injection

A lipid mixture of POPC, POPS, DSPE-PEG-biotin, and 1,2-dipalmitoyl-sn-glycero-3-phosphoethanolamine (DPPE)-rhodamine (78.9:20:1:0.1 mol%) was prepared in chloroform. GUVs filled with protein buffer were grown by the gel-assisted method [60]. Briefly, a 5% (w/w) polyvinyl alcohol (PVA) solution was prepared in protein buffer (25 mM tris-HCl, pH 7.4, 150 mM NaCl). The PVA solution was spread on a microscope coverslip and then dried for at least 30 min at 50°C. 10–15 μl of lipids dissolved in chloroform (1 mg/ml) were spread on the dried PVA film and placed under vacuum for 1 h to eliminate the solvent. A chamber was formed with a homemade Teflon spacer sandwiched between two microscope slides and filled with protein buffer for 10 min at RT. Then, GUVs were harvested by gentle tapping on the bottom of the chamber and collected using a micropipette without touching the PVA film to avoid sample contamination. To immobilize GUVs, cleaned coverslips were incubated for 20 min at RT with a 1:1 mixture of 1 mg/ml BSA-biotin and 1 mg/ml BSA (both diluted in protein buffer to maintain osmolarity), following the protocol from [61]. After incubation, coverslips were washed with distilled water and incubated with 0.005 mg/ml avidin. Subsequently, slides were washed and dried with N_2_. These coverslips were used to assemble a homemade observation chamber using a Teflon spacer, where GUVs were deposited and let to settle down for at least 10 min.

The micropipettes used to perform the injection were fabricated from thin wall borosilicate glass capillaries with filament (Harvard Apparatus, Holliston, MA, USA) in a pipette puller (Sutter Instruments, Novato, CA, USA) to obtain bee-needle type tips. For the injection experiments, immobilized GUVs were imaged under a Leica TCS SP5 confocal microscope. The micropipette was placed on a mechanical holder attached to a micromanipulator (Sutter Instruments) and then connected to a Femtojet microinjector set (Eppendorf). Injection was performed in a 15° angle, using a pressure of injection of 150 hPa, time of injection of 5.0 s and a compensation pressure of 1 hPa. The solution injected corresponded to a 4× protein mixture stock (2.4 mM PfBro1 and 4.8 mM of either PfVps32 or PfVps60 dissolved in 1× buffer) or its individual components, and 0.03 mg/ml PEG-FITC to monitor injection.

### Generation of *PfVps60* KO strain

Homology regions (HR) of the 5’UTR (HR1, spanning positions −762 to −243 from the *PfVps60* start codon) and 3’UTR (HR2, spanning positions 1,012 to 1,553 from the *PfVps60* start codon) were PCR amplified using genomic DNA purified from a *P*. *falciparum* 3D7 culture synchronized at late stages. Primers used for PCR amplification are listed in S2 table. The generated HR1 and HR2 were cloned by ligation using restriction sites *SpeI* and *AflII* (HR1), and *EcoRI* and *NcoI* (HR2) into a modified pL6-*egfp* donor plasmid [62] in which the *yfcu* cassette had been removed [63]. The single guide RNA (sgRNA) specific for the *PfVps60* gene and targeting the sequence near the 5’ end (sgRNA 5’, position −225, −206) was generated by cloning annealed oligonucleotides into the *BtgZI* site to generate the pL7-*pfvps60*_KO_sgRNA5’ plasmid. On the other hand, the pDC2-Cas9-U6-h*dhfr* vector [64] was modified by cloning a sgRNA specific for the sequence near the 3’ end (sgRNA 3’, position 980, 999) into the *Btg*ZI site of this plasmid to generate the pDC2-Cas9-U6*-pfvps60*_KO_sgRNA3’ plasmid. All guides were cloned using the In-Fusion system (Clontech, Japan).

For transfection of 3D7 rings, 60 μg of circular pDC2-Cas9-U6-h*dhfr-pfvps60*_KO_sgRNA3’ plasmid and 30 μg of linearized (with *PvuI*) donor plasmid were precipitated, washed and resuspended in 30 μl of sterile 10 mM tris, 1 mM EDTA (TE) buffer. Then, plasmids were diluted in 370 μl of Cytomix buffer (120 mM KCl, 0.15 mM CaCl_2_, 10mM K_2_HPO_4_/KH_2_PO_4_, 25 mM Hepes, 2 mM EGTA, 5 mM MgCl_2,_ pH 7.6) and introduced into parasites by electroporation using a Bio-Rad Gene Pulser Xcell system, at 310 V, 950 μF of capacitance and without resistance. Electroporated parasites were carefully recovered and resuspended in RPMI medium supplemented with 0.5% (w/v) Albumax II and 2 mM L-glutamine. Twenty-four hours after transfection, cultures were selected with 10 nM WR99210 for 4 consecutive days [65]. To validate the integration of the plasmids, a diagnostic PCR analysis was performed using LA Taq DNA polymerase (Takara, Japan), the primers listed in S2 Table and gDNA obtained from the *PfVps60* KO strain, and compared with the WT 3D7 strain. The fitness of the generated line, compared with the parental 3D7 line, was evaluated by calculating the percentage of newly formed rings in tightly synchronized cultures. Initial parasitemia was determined at ~18 hpi, then rings parasitemia was determined at different time points within the period where most schizont bursting and reinvasion events occurred (44 to 62 hpi). The final point was 74 hpi when all viable schizonts had burst. Data points were determined by the proportion of rings relative to the total number of rings at the end of the assay. Data was fitted to a sigmoidal dose-response curve and the time to generate 50% or the rings in each population was determined [66].

### EVs purification

For purification of EVs, medium was collected at 40 hpi from pRBC cultures with an initial parasitemia of 3%. EV isolation was performed as previously described [67]. Briefly, samples were prepared by sequential centrifugations of conditioned medium to remove large aggregates. First, cultures were centrifuged for 10 min at 400× g, the cell pellet was discarded, and supernatant was further centrifuged at 2,000× g for 10 min twice. In both cases, a small pellet was discarded. Finally, 25 ml of the supernatant was placed in an Amicon Ultra-15 centrifugal filter (100 kDa cut-off, Millipore-Merck, Cork, Ireland) and centrifuged for 20 min at 3,400× g. One ml of the resulting concentrated solution was collected and transferred to a 10 ml homemade Sepharose CL-4B column previously equilibrated with PBS. EV purification was performed by gravity flow at RT and 0.5-ml fractions were collected. EVs were enriched in fractions 8 and 9, which were combined, concentrated and resuspended to a final volume of 200 μl to be used for the rest of EV characterization experiments.

### EV size and abundancy measurements

Dynamic light scattering was used to measure particle size in the purified EVs population as described before [68]. In order to obtain the optimum light scattering intensity, 100 μl of purified EVs were resuspended in 900 μl of filtered (0.22 μm) PBS diluted 1:2 in 4% paraformaldehyde (PFA). Mean particle size of vesicle dispersions and the derived count rate (Kilo counts per second, kcps) were determined in triplicates from light diffusion measured at 25°C and an attenuator index of 8, using Zetasizer Nano S (Malvern Instruments, Ltd., Malvern, UK).

### EVs imaging

For STORM visualization of purified EVs, the protocol for super-resolution microscopy of vaccinia virus particles described by Gray & Albrecht [69] was followed. Briefly, a clean coverslip was washed 3 times with ethanol, acetone and deionized water sequentially. Then, coverslips were sonicated in 1 M KOH for 20 min at RT, washed thoroughly with water and placed in a 12-well plate. Next, the purified EVs were sonicated for 30 s to avoid aggregation, diluted in 1 mM tris-HCl, pH 9.0, and deposited on the cleaned coverslips. EVs were left to adhere for 60 min at RT and the rest of the solution was carefully removed with a pipette. The bound EVs were then fixed with 4% PFA for 15 min at RT, washed and incubated in quenching buffer (0.25% NH_4_Cl in PBS) for 5 min at RT. After this time, EV-coated coverslips were incubated with blocking solution (5% BSA in PBS) for 30 min at RT and then, with either anti-PfVps32, anti-PfVps60 or anti-PfBro1 polyclonal antibodies labeled with Alexa Fluor 647 (1:100 in all cases) to detect EVs from parasite origin, or anti-GPA, (1:100), followed by anti-rabbit Alexa Fluor 488 (1:100), to reveal EVs derived from the RBC plasma membrane. Finally, coverslips were mounted with OxEA buffer for STORM imaging as described previously.

## Supporting information

S1 Table Snf-7 domain-containing proteins encoded in the *P*. *falciparum* genome. Modified from [23]. Percentages of similarity (S), identity (I) and expectation value (*E*-value) relative to *P*. *falciparum* proteins were determined using the Expert Protein Analysis Systems (ExPASy) Proteomics Server by the NCBI BLAST service program.
(TIF)

S2 Table Primers used for CRISPR/Cas9 gene edition. (TIF)

S1 Fig Multiple sequence alignment for Bro1-homologues in yeast (Bro1), *P*. *falciparum* (PfBro1) and humans (Alix). Conserved residues are shadowed in blue. Conserved amino acids present in Bro1-containing proteins are indicated with colored circles, yellow for the polar cluster I and green for the polar cluster II. A key isoleucine involved in Vps32 binding is indicated with a star. The conserved PEXEL motif is double underlined in pink. The secondary structure for PfBro1 is displayed below the sequences, alpha helices represented in red and beta-sheets in green. Sequence alignments were performed with Clustal Omega and edited in Jalview 2.
(TIF)

S2 Fig Tertiary prediction and membrane orientation of PfBro1. The hydrophobic tail is colored in pink. Outer membrane leaflet is colored in red, inner leaflet in blue. The structure was generated using the Phyre2 server and the OPM database.
(TIF)

S3 Fig Purification of proteins and antibody validation. Left panels: SDS-PAGE gels stained with Coomassie blue showing the purified fractions of **(a)** PfVps32, **(b)** PfVps60 and **(c)** PfBro1 that were used for this study. The rest of the panels show Western blot assays of the induced bacterial lysates (r) or *P*. *falciparum-*infected RBCs (*P*.*f*.) at 30 hours post invasion, using the specific antibodies or preimmune serum (PS) as indicated above the corresponding panels. Arrows indicate the approximate molecular weight. The polyacrylamide percentage is indicated below each gel.
(TIF)

S4 Fig Expression of PfVps32, PfVps60 and PfBro1 during the *P*. *falciparum* intraerythrocytic cycle. Western blot analysis of *P*. *falciparum* in saponin, Triton X-100 or RIPA buffer protein extracts at different hours post invasion (indicated above the upper panels) to monitor protein expression of **(a)** PfVps32, **(b)** PfVps60 and **(c)** PfBro1. Arrows indicate the approximate molecular weight. IB: antibody used for loading control in the lower panels.
(TIF)

S5 Fig Antibody validation for fluorescence confocal microscopy assays. Human erythrocytes were infected with *P*. *falciparum* and fixed with 4% PFA. **(a)** PfVps32, PfVps60 or PfBro1 (green) and WGA (red) were detected by indirect confocal immunofluorescence microscopy using the corresponding specific antibodies. Non-infected red blood cells did not show any antibody recognition. As negative controls, cells were incubated with **(b)** IgGs purified from preimmune serum (PS) or **(c)** only the secondary antibody anti-rabbit-Alexa488. Cell nuclei were visualized with Hoechst 33342 (blue). Scale bar: 10 μm.
(TIF)

S6 Fig Incubation of PfBro1t-OG488 with GUVs. POPC:POPS (80:20) GUVs labeled with DiI_C18_ were diluted in protein buffer, incubated with 600 nM of PfBro1t in a 1:3 ratio (labeled:unlabeled protein), and visualized by fluorescence confocal microscopy.
(TIF)

S7 Fig Protein docking of PfBro1 and PfVps60. Predicted structure of **(a)** PfVps60 in its auto-inhibited form and **(b)** Bro1-domain of PfBro1. **(c)** Protein docking simulation showing the PfVps60 “opening”. All images were generated using PyMOL.
(TIF)

S8 Fig Representative images of a GUV selected for injection. Panels show the top and side view of a typical vesicle selected to perform protein injection.
(TIF)

S9 Fig Femtoliter injection of PEG-FITC in protein buffer. GUVs composed by POPC:POPS:DSPE-biotin (79:20:1) and labeled with DPPE-rhodamine (0.1 mol%) were grown on a PVA substrate using protein buffer, harvested after 10 min and deposited on an avidin-coated coverslip, and injected with PEG-FITC. No alterations were observed up to 5 min after injection.
(TIF)

S1 Video Z-stack reconstruction by fluorescence confocal microscopy of POPC:POPS (80:20) GUVs labeled with DiI_C18_ (0.1 mol%) incubated with 600 nM PfBro1 and 1200 nM PfVps60. Intraluminal and interconnected buds of different sizes and some tubular structures are observed.
(AVI)

S2 Video Time-lapse fluorescence confocal microscopy of the injection of POPC:POPS:DSPE-biotin (79:20:1) GUVs labeled with DPPE-rhodamine (0.1 mol%) with free PEG-FITC. The video is accelerated 4.5×.
(AVI)

S3 Video Time-lapse fluorescence confocal microscopy of the injection of POPC:POPS:DSPE-biotin (79:20:1) GUVs labeled with DPPE-rhodamine (0.1 mol%) with a mixture of PfBro1:PfVps32 (1:2) together with PEG-FITC. The video is accelerated 4.5×.
(AVI)

S1 Supporting Materials and Methods Supporting Materials and Methods. (PDF)

## References

[1] WHO. World Malaria Report 2019 Genova: World Health Organization; 2019. 232 pp]. Available from: https://www.who.int/malaria/publications/world-malaria-report-2019/en/.

[2] Couper KN, Barnes T, Hafalla JCR, Combes V, Ryffel B, Secher T, et al. Parasite-Derived Plasma Microparticles Contribute Significantly to Malaria Infection-Induced Inflammation through Potent Macrophage Stimulation. Plos Pathogens. 2010;6(1). ARTN doi: 10.1371/journal.ppat.1000744 WOS:000274227100034. 20126448 PMC2813278

[3] Combes V, Taylor TE, Juhan-Vague I, Mege JL, Mwenechanya J, Tembo M, et al. Circulating endothelial microparticles in Malawian children with severe falciparum malaria complicated with coma. Jama-Journal of the American Medical Association. 2004;291(21):2542–4. doi: 10.1001/jama.291.21.2542-b WOS:000221738800014. 15173142

[4] Mfonkeu JBP, Gouado I, Kuate HF, Zambou O, Zollo PHA, Grau GER, et al. Elevated Cell-Specific Microparticles Are a Biological Marker for Cerebral Dysfunctions in Human Severe Malaria. Plos One. 2010;5(10). ARTN doi: 10.1371/journal.pone.0013415 WOS:000282941000026. PMC2954805 20976232

[5] Campos FMF, Franklin BS, Teixeira-Carvalho A, Filho ALS, de Paula SCO, Fontes CJ, et al. Augmented plasma microparticles during acute Plasmodium vivax infection. Malaria Journal. 2010;9. Artn 32710.1186/1475-2875-9-327. WOS:000287600800002. doi: 10.1186/1475-2875-9-9 21080932 PMC2998527

[6] Lauer SA, Rathod PK, Ghori N, Haldar K. A membrane network for nutrient import in red cells infected with the malaria parasite. Science. 1997;276(5315):1122–5. doi: 10.1126/science.276.5315.1122 WOS:A1997WZ22500047. 9148808

[7] Akers JC, Gonda D, Kim R, Carter BS, Chen CC. Biogenesis of extracellular vesicles (EV): exosomes, microvesicles, retrovirus-like vesicles, and apoptotic bodies. Journal of Neuro-Oncology. 2013;113(1):1–11. doi: 10.1007/s11060-013-1084-8 WOS:000318300700001. 23456661 PMC5533094

[8] Schlacht A, Herman EK, Klute MJ, Field MC, Dacks JB. Missing Pieces of an Ancient Puzzle: Evolution of the Eukaryotic Membrane-Trafficking System. Cold Spring Harbor Perspectives in Biology. 2014;6(10). ARTN a01604810.1101/cshperspect.a016048. WOS:000346448700007. doi: 10.1101/cshperspect.a016048 25274701 PMC4176009

[9] van Niel G D’Angelo G, Raposo G. Shedding light on the cell biology of extracellular vesicles. Nat Rev Mol Cell Bio. 2018;19(4):213–28. doi: 10.1038/nrm.2017.125 WOS:000427924900008. 29339798

[10] Raposo G, Stoorvogel W. Extracellular vesicles: exosomes, microvesicles, and friends. J Cell Biol. 2013;200(4):373–83. doi: 10.1083/jcb.201211138 ; PubMed Central PMCID: PMC3575529. 23420871 PMC3575529

[11] Winter V, Hauser MT. Exploring the ESCRTing machinery in eukaryotes. Trends Plant Sci. 2006;11(3):115–23. Epub 2006/02/21. S1360-1385(06)00034-3 [pii]doi: 10.1016/j.tplants.2006.01.008 ; PubMed Central PMCID: PMC2865992. 16488176 PMC2865992

[12] Katzmann DJ, Odorizzi G, Emr SD. Receptor downregulation and multivesicular-body sorting. Nat Rev Mol Cell Biol. 2002;3(12):893–905. Epub 2002/12/04. doi: 10.1038/nrm973 [pii]. . 12461556

[13] Raiborg C, Stenmark H. The ESCRT machinery in endosomal sorting of ubiquitylated membrane proteins. Nature. 2009;458(7237):445–52. doi: 10.1038/nature07961 WOS:000264532400033. 19325624

[14] Katzmann DJ, Stefan CJ, Babst M, Emr SD. Vps27 recruits ESCRT machinery to endosomes during MVB sorting. Journal of Cell Biology. 2003;162(3):413–23. doi: 10.1083/jcb.200302136 WOS:000184667900007. 12900393 PMC2172707

[15] Gill DJ, Teo H, Sun J, Perisic O, Veprintsev DB, Emr SD, et al. Structural insight into the ESCRT-I/-II link and its role in MVB trafficking. Embo Journal. 2007;26(2):600–12. doi: 10.1038/sj.emboj.7601501 WOS:000243730700030. 17215868 PMC1783442

[16] Im YJ, Wollert T, Boura E, Hurley JH. Structure and Function of the ESCRT-II-III Interface in Multivesicular Body Biogenesis. Developmental Cell. 2009;17(2):234–43. doi: 10.1016/j.devcel.2009.07.008 WOS:000269138600013. 19686684 PMC2749878

[17] Boura E, Rozycki B, Chung HS, Herrick DZ, Canagarajah B, Cafiso DS, et al. Solution Structure of the ESCRT-I and -II Supercomplex: Implications for Membrane Budding and Scission. Structure. 2012;20(5):874–86. doi: 10.1016/j.str.2012.03.008 WOS:000304214400013. 22579254 PMC3572543

[18] Babst M, Katzmann DJ, Snyder WB, Wendland B, Emr SD. Endosome-associated complex, ESCRT-II, recruits transport machinery for protein sorting at the multivesicular body. Developmental Cell. 2002;3(2):283–9. doi: 10.1016/s1534-5807(02)00219-8 WOS:000177325700017. 12194858

[19] Teis D, Saksena S, Judson BL, Emr SD. ESCRT-II coordinates the assembly of ESCRT-III filaments for cargo sorting and multivesicular body vesicle formation. Embo Journal. 2010;29(5):871–83. doi: 10.1038/emboj.2009.408 WOS:000275169800002. 20134403 PMC2837172

[20] Obita T, Saksena S, Ghazi-Tabatabai S, Gill DJ, Perisic O, Emr SD, et al. Structural basis for selective recognition of ESCRT-III by the AAA ATPase Vps4. Nature. 2007;449(7163):735–U11. doi: 10.1038/nature06171 WOS:000250045000047. 17928861

[21] Hurley JH. ESCRTs are everywhere. Embo J. 2015;34(19):2398–407. doi: 10.15252/embj.201592484 WOS:000362457800006. 26311197 PMC4601661

[22] Henne WM, Stenmark H, Emr SD. Molecular mechanisms of the membrane sculpting ESCRT pathway. Cold Spring Harb Perspect Biol. 2013;5(9). Epub 2013/09/05. doi: 10.1101/cshperspect.a016766 [pii]5/9/a016766 [pii]. 24003212. 24003212 PMC3753708

[23] Leung KF, Dacks JB, Field MC. Evolution of the multivesicular body ESCRT machinery; retention across the eukaryotic lineage. Traffic. 2008;9(10):1698–716. doi: 10.1111/j.1600-0854.2008.00797.x WOS:000259238000013. 18637903

[24] Dores MR, Paing MM, Lin H, Montagne WA, Marchese A, Trejo J. AP-3 regulates PAR1 ubiquitin-independent MVB/lysosomal sorting via an ALIX-mediated pathway. Mol Biol Cell. 2012;23(18):3612–23. Epub 2012/07/27. doi: 10.1091/mbc.E12-03-0251 [pii]. ; PubMed Central PMCID: PMC3442409. 22833563 PMC3442409

[25] Jimenez-Ruiz E, Morlon-Guyot J, Daher W, Meissner M. Vacuolar protein sorting mechanisms in apicomplexan parasites. Molecular and Biochemical Parasitology. 2016;209(1–2):18–25. doi: 10.1016/j.molbiopara.2016.01.007 WOS:000390632200004. 26844642 PMC5154328

[26] Yang M, Coppens I, Wormsley S, Baevova P, Hoppe HC, Joiner KA. The Plasmodium falciparum Vps4 homolog mediates multivesicular body formation. Journal of Cell Science. 2004;117(17):3831–8. doi: 10.1242/jcs.01237 WOS:000223733100013. 15252121

[27] Winter V, Hauser MT. Exploring the ESCRTing machinery in eukaryotes. Trends in Plant Science. 2006;11(3):115–23. doi: 10.1016/j.tplants.2006.01.008 WOS:000236648200004. 16488176 PMC2865992

[28] Kim JW, Sitaraman S, Hierro A, Beach BM, Odorizzi G, Hurley JH. Structural basis for endosomal targeting by the Bro1 domain. Developmental Cell. 2005;8(6):937–47. doi: 10.1016/j.devcel.2005.04.001 WOS:000230006200017. 15935782 PMC2862258

[29] Pick C, Ebersberger I, Spielmann T, Bruchhaus I, Burmester T. Phylogenomic analyses of malaria parasites and evolution of their exported proteins. BMC Evol Biol. 2011;11:167. Epub 2011/06/17. doi: 10.1186/1471-2148-11-167 ; PubMed Central PMCID: PMC3146879. 21676252 PMC3146879

[30] Mu R, Dussupt V, Jiang J, Sette P, Rudd V, Chuenchor W, et al. Two distinct binding modes define the interaction of Brox with the C-terminal tails of CHMP5 and CHMP4B. Structure. 2012;20(5):887–98. Epub 2012/04/10. doi: 10.1016/j.str.2012.03.001 ; PubMed Central PMCID: PMC3350598. 22484091 PMC3350598

[31] Pisitkun T, Shen RF, Knepper MA. Identification and proteomic profiling of exosomes in human urine. Proc Natl Acad Sci U S A. 2004;101(36):13368–73. Epub 2004/08/25. doi: 10.1073/pnas.0403453101 ; PubMed Central PMCID: PMC516573. 15326289 PMC516573

[32] Babst M, Katzmann DJ, Estepa-Sabal EJ, Meerloo T, Emr SD. Escrt-III: an endosome-associated heterooligomeric protein complex required for mvb sorting. Dev Cell. 2002;3(2):271–82. Epub 2002/08/27. doi: 10.1016/s1534-5807(02)00220-4 . 12194857

[33] Avalos-Padilla Y, Knorr RL, Javier-Reyna R, Garcia-Rivera G, Lipowsky R, Dimova R, et al. The Conserved ESCRT-III Machinery Participates in the Phagocytosis of Entamoeba histolytica. Frontiers in Cellular and Infection Microbiology. 2018;8. ARTN 5310.3389/fcimb.2018.00053. WOS:000426386400001. doi: 10.3389/fcimb.2018.00053 29546036 PMC5838018

[34] Wollert T, Hurley JH. Molecular mechanism of multivesicular body biogenesis by ESCRT complexes. Nature. 2010;464(7290):864–U73. doi: 10.1038/nature08849 WOS:000276397300031. 20305637 PMC2851844

[35] Virtanen JA, Cheng KH, Somerharju P. Phospholipid composition of the mammalian red cell membrane can be rationalized by a superlattice model. P Natl Acad Sci USA. 1998;95(9):4964–9. doi: 10.1073/pnas.95.9.4964 WOS:000073415700034. 9560211 PMC20196

[36] Bajorek M, Schubert HL, McCullough J, Langelier C, Eckert DM, Stubblefield WMB, et al. Structural basis for ESCRT-III protein autoinhibition. Nature Structural & Molecular Biology. 2009;16(7):754–U95. doi: 10.1038/nsmb.1621 WOS:000267764500014. 19525971 PMC2712734

[37] Tang SG, Buchkovich NJ, Henne WM, Banjade S, Kim YJ, Emr SD. ESCRT-III activation by parallel action of ESCRT-I/II and ESCRT-0/Bro1 during MVB biogenesis. Elife. 2016;5. ARTN e1550710.7554/eLife.15507. WOS:000376391800001. doi: 10.7554/eLife.15507 27074665 PMC4865371

[38] Colombo M, Raposo G, Thery C. Biogenesis, Secretion, and Intercellular Interactions of Exosomes and Other Extracellular Vesicles. Annual Review of Cell and Developmental Biology, Vol 30. 2014;30:255–89. doi: 10.1146/annurev-cellbio-101512-122326 WOS:000348434900012. 25288114

[39] Matz JM, Beck JR, Blackman MJ. The parasitophorous vacuole of the blood-stage malaria parasite. Nat Rev Microbiol. 2020;18(7):379–91. Epub 2020/01/26. doi: 10.1038/s41579-019-0321-3 . 31980807

[40] Hiller NL, Bhattacharjee S, van Ooij C, Liolios K, Harrison T, Lopez-Estrano C, et al. A host-targeting signal in virulence proteins reveals a secretome in malarial infection. Science. 2004;306(5703):1934–7. Epub 2004/12/14. doi: 10.1126/science.1102737 . 15591203

[41] Chang HH, Falick AM, Carlton PM, Sedat JW, DeRisi JL, Marletta MA. N-terminal processing of proteins exported by malaria parasites. Mol Biochem Parasitol. 2008;160(2):107–15. Epub 2008/06/07. doi: 10.1016/j.molbiopara.2008.04.011 ; PubMed Central PMCID: PMC2922945. 18534695 PMC2922945

[42] Sampaio NG, Emery SJ, Garnham AL, Tan QY, Sisquella X, Pimentel MA, et al. Extracellular vesicles from early stage Plasmodium falciparum-infected red blood cells contain PfEMP1 and induce transcriptional changes in human monocytes. Cell Microbiol. 2018;20(5):e12822. Epub 2018/01/20. doi: 10.1111/cmi.12822 . 29349926

[43] Regev-Rudzki N, Wilson DW, Carvalho TG, Sisquella X, Coleman BM, Rug M, et al. Cell-Cell Communication between Malaria-Infected Red Blood Cells via Exosome-like Vesicles. Cell. 2013;153(5):1120–33. doi: 10.1016/j.cell.2013.04.029 WOS:000319456800018. 23683579

[44] Babatunde KA, Subramanian BY, Ahouidi AD, Murillo PM, Walch M, Mantel PY. Role of Extracellular Vesicles in Cellular Cross Talk in Malaria. Front Immunol. 2020;11. ARTN 22 doi: 10.3389/fimmu.2020.00022 WOS:000512689100001. 32082312 PMC7005784

[45] Colombo M, Moita C, van Niel G, Kowal J, Vigneron J, Benaroch P, et al. Analysis of ESCRT functions in exosome biogenesis, composition and secretion highlights the heterogeneity of extracellular vesicles. Journal of Cell Science. 2013;126(24):5553–65. doi: 10.1242/jcs.128868 WOS:000328686600003. 24105262

[46] Booth AM, Fang Y, Fallon JK, Yang JM, Hildreth JEK, Gould SJ. Exosomes and HIV Gag bud from endosome-like domains of the T cell plasma membrane. Journal of Cell Biology. 2006;172(6):923–35. doi: 10.1083/jcb.200508014 WOS:000235971900017. 16533950 PMC2063735

[47] Odorizzi G, Katzmann DJ, Babst M, Audhya A, Emr SD. Bro1 is an endosome-associated protein that functions in the MVB pathway in Saccharomyces cerevisiae. Journal of Cell Science. 2003;116(10):1893–903. doi: 10.1242/jcs.00395 WOS:000183098900005. 12668726

[48] Goldberg DE. Plasmodium protein export at higher PEXEL resolution. Cell Host Microbe. 2012;12(5):609–10. Epub 2012/11/20. doi: 10.1016/j.chom.2012.11.001 . 23159050

[49] Booth A, Marklew CJ, Ciani B, Beales PA. In Vitro Membrane Remodeling by ESCRT is Regulated by Negative Feedback from Membrane Tension. Iscience. 2019;15:173–+. doi: 10.1016/j.isci.2019.04.021 WOS:000470104600016. 31060000 PMC6503128

[50] Mierzwa BE, Chiaruttini N, Redondo-Morata L, von Filseck JM, Konig J, Larios J, et al. Dynamic subunit turnover in ESCRT-III assemblies is regulated by Vps4 to mediate membrane remodelling during cytokinesis. Nature Cell Biology. 2017;19(7):787–+. doi: 10.1038/ncb3559 WOS:000404408800011. 28604678 PMC5493987

[51] Adell MAY, Migliano SM, Upadhyayula S, Bykov YS, Sprenger S, Pakdel M, et al. Recruitment dynamics of ESCRT-III and Vps4 to endosomes and implications for reverse membrane budding. Elife. 2017;6. ARTN e3165210.7554/eLife.31652. WOS:000414139400001. doi: 10.7554/eLife.31652 29019322 PMC5665648

[52] Morita E, Sandrin V, Chung HY, Morham SG, Gygi SP, Rodesch CK, et al. Human ESCRT and ALIX proteins interact with proteins of the midbody and function in cytokinesis. Embo Journal. 2007;26(19):4215–27. doi: 10.1038/sj.emboj.7601850 WOS:000250466800004. 17853893 PMC2230844

[53] Piper RC, Cooper AA, Yang H, Stevens TH. VPS27 controls vacuolar and endocytic traffic through a prevacuolar compartment in Saccharomyces cerevisiae. J Cell Biol. 1995;131(3):603–17. Epub 1995/11/01. doi: 10.1083/jcb.131.3.603 ; PubMed Central PMCID: PMC2120612. 7593183 PMC2120612

[54] Raymond CK, Howald-Stevenson I, Vater CA, Stevens TH. Morphological classification of the yeast vacuolar protein sorting mutants: evidence for a prevacuolar compartment in class E vps mutants. Mol Biol Cell. 1992;3(12):1389–402. Epub 1992/12/01. doi: 10.1091/mbc.3.12.1389 ; PubMed Central PMCID: PMC275707. 1493335 PMC275707

[55] Matthews KM, Pitman EL, de Koning-Ward TF. Illuminating how malaria parasites export proteins into host erythrocytes. Cell Microbiol. 2019;21(4):e13009. Epub 2019/01/19. doi: 10.1111/cmi.13009 . 30656810

[56] Mantel PY, Hoang AN, Goldowitz I, Potashnikova D, Hamza B, Vorobjev I, et al. Malaria-Infected Erythrocyte-Derived Microvesicles Mediate Cellular Communication within the Parasite Population and with the Host Immune System. Cell Host & Microbe. 2013;13(5):521–34. doi: 10.1016/j.chom.2013.04.009 WOS:000330850800006. 23684304 PMC3687518

[57] Lambros C, Vanderberg JP. Synchronization of Plasmodium-Falciparum Erythrocytic Stages in Culture. Journal of Parasitology. 1979;65(3):418–20. doi: 10.2307/3280287 WOS:A1979HK51700015. 383936

[58] Mehnert AK, Simon CS, Guizetti J. Immunofluorescence staining protocol for STED nanoscopy of Plasmodium-infected red blood cells. Molecular and Biochemical Parasitology. 2019;229:47–52. doi: 10.1016/j.molbiopara.2019.02.007 WOS:000465052800006. 30831155

[59] Nahidiazar L, Agronskaia AV, Broertjes J, van den Broek B, Jalink K. Optimizing Imaging Conditions for Demanding Multi-Color Super Resolution Localization Microscopy. Plos One. 2016;11(7). ARTN e015888410.1371/journal.pone.0158884. WOS:000380005400149. doi: 10.1371/journal.pone.0158884 27391487 PMC4938622

[60] Weinberger A, Tsai FC, Koenderink GH, Schmidt TF, Itri R, Meier W, et al. Gel-assisted formation of giant unilamellar vesicles. Biophys J. 2013;105(1):154–64. Epub 2013/07/05. doi: 10.1016/j.bpj.2013.05.024 ; PubMed Central PMCID: PMC3699747. 23823234 PMC3699747

[61] Maan R, Loiseau E, Bausch AR. Adhesion of Active Cytoskeletal Vesicles. Biophys J. 2018;115(12):2395–402. Epub 2018/11/21. doi: 10.1016/j.bpj.2018.10.013 PubMed Central PMCID: PMC6301914. 30455042 PMC6301914

[62] Ghorbal M, Gorman M, Macpherson CR, Martins RM, Scherf A, Lopez-Rubio JJ. Genome editing in the human malaria parasite Plasmodium falciparum using the CRISPR-Cas9 system. Nat Biotechnol. 2014;32(8):819–21. Epub 2014/06/02. doi: 10.1038/nbt.2925 24880488

[63] Llora-Batlle O, Michel-Todo L, Witmer K, Toda H, Fernandez-Becerra C, Baum J, et al. Conditional expression of PfAP2-G for controlled massive sexual conversion in Plasmodium falciparum. Sci Adv. 2020;6(24):eaaz5057. Epub 2020/06/25. doi: 10.1126/sciadv.aaz5057 ; PubMed Central PMCID: PMC7286680. 32577509 PMC7286680

[64] Lim MY, LaMonte G, Lee MCS, Reimer C, Tan BH, Corey V, et al. UDP-galactose and acetyl-CoA transporters as Plasmodium multidrug resistance genes. Nat Microbiol. 2016;1:16166. Epub 2016/09/20. doi: 10.1038/nmicrobiol.2016.166 ; PubMed Central PMCID: PMC5575994. 27642791 PMC5575994

[65] Knuepfer E, Napiorkowska M, van Ooij C, Holder AA. Generating conditional gene knockouts in Plasmodium—a toolkit to produce stable DiCre recombinase-expressing parasite lines using CRISPR/Cas9. Sci Rep. 2017;7(1):3881. Epub 2017/06/22. doi: 10.1038/s41598-017-03984-3 ; PubMed Central PMCID: PMC5478596. 28634346 PMC5478596

[66] Rovira-Graells N, Gupta AP, Planet E, Crowley VM, Mok S, Ribas de Pouplana L, et al. Transcriptional variation in the malaria parasite Plasmodium falciparum. Genome Res. 2012;22(5):925–38. Epub 2012/03/15. doi: 10.1101/gr.129692.111 ; PubMed Central PMCID: PMC3337437. 22415456 PMC3337437

[67] Borgheti-Cardoso LN, Kooijmans SAA, Chamorro LG, Biosca A, Lantero E, Ramirez M, et al. Extracellular vesicles derived from Plasmodium-infected and non-infected red blood cells as targeted drug delivery vehicles. Int J Pharm. 2020;587:119627. Epub 2020/07/13. doi: 10.1016/j.ijpharm.2020.119627 . 32653596

[68] Hubert A, Subra C, Jenabian MA, Tremblay Labrecque PF, Tremblay C, Laffont B, et al. Elevated Abundance, Size, and MicroRNA Content of Plasma Extracellular Vesicles in Viremic HIV-1+ Patients: Correlations With Known Markers of Disease Progression. J Acquir Immune Defic Syndr. 2015;70(3):219–27. Epub 2015/07/17. doi: 10.1097/QAI.0000000000000756 ; PubMed Central PMCID: PMC4627170. 26181817 PMC4627170

[69] Gray R, Albrecht D. Super-resolution Microscopy of Vaccinia Virus Particles. Methods Mol Biol. 2019;2023:255–68. Epub 2019/06/27. doi: 10.1007/978-1-4939-9593-6_16 . 31240683

