# Correction: The ESCRT-III machinery participates in the production of extracellular vesicles and protein export during *Plasmodium falciparum* infection

**DOI:** 10.1371/journal.ppat.1013606

**Published:** 2025-10-22

**Authors:** Yunuen Avalos-Padilla, Vasil N. Georgiev, Elena Lantero, Silvia Pujals, René Verhoef, Livia N. Borgheti-Cardoso, Lorenzo Albertazzi, Rumiana Dimova, Xavier Fernàndez-Busquets

The Data Availability statement for this article is incorrect. The correct statement is: All relevant data are within the manuscript and its Supporting Information files. Raw, individual-level data underlying the graphs and figures are provided in the Supporting Information file S1 Data.

The raw data underlying graphs and figures are missing from the list of Supporting Information. The authors have provided the data as Supporting Information file S1 Data. With this correction [1], all relevant data are now provided.

## Supporting information

S1 Data (XLSX)
