# Supplementary figures and images for: The ESCRT-III machinery participates in the production of extracellular vesicles and protein export during Plasmodium falciparum infection
Source: PLoS Pathog. 2021 Apr 2;17(4):e1009455. doi: 10.1371/journal.ppat.1009455 (PMC9159051; doi:10.1371/journal.ppat.1009455)

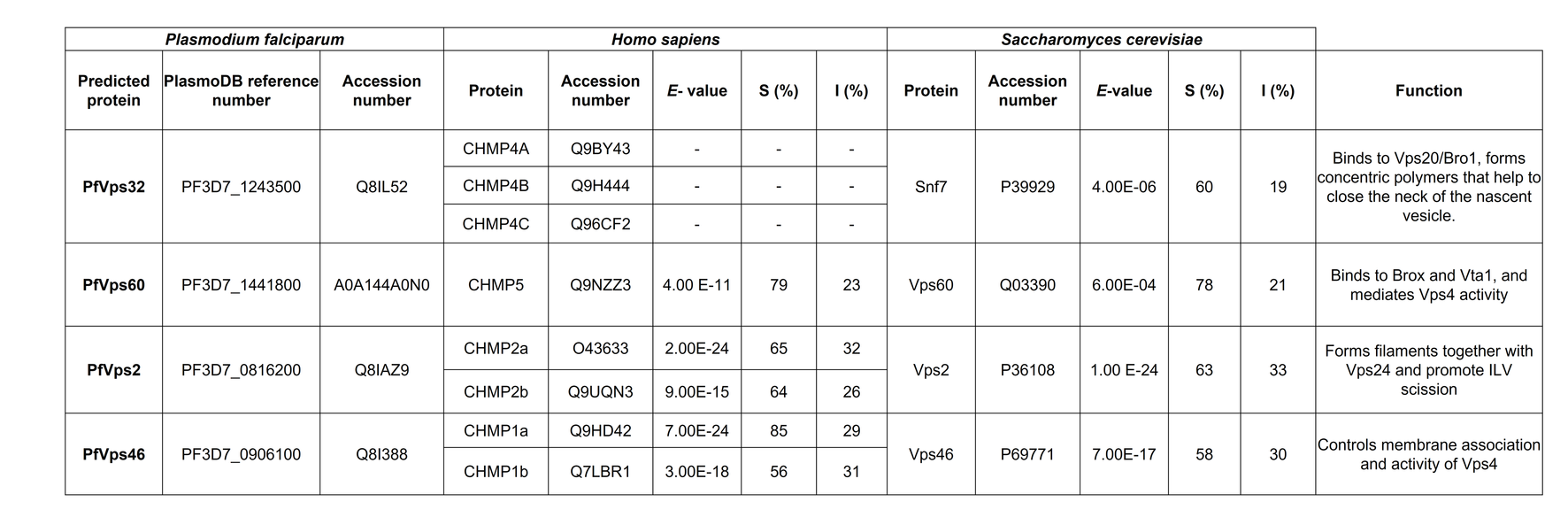

Supplement: S1 Table — Modified from [23]. Percentages of similarity (S), identity (I) and expectation value (E-value) relative to P. falciparum proteins were determined using the Expert Protein Analysis Systems (ExPASy) Proteomics Server by the NCBI BLAST service program. (TIF) [file ppat.1009455.s001.tif]

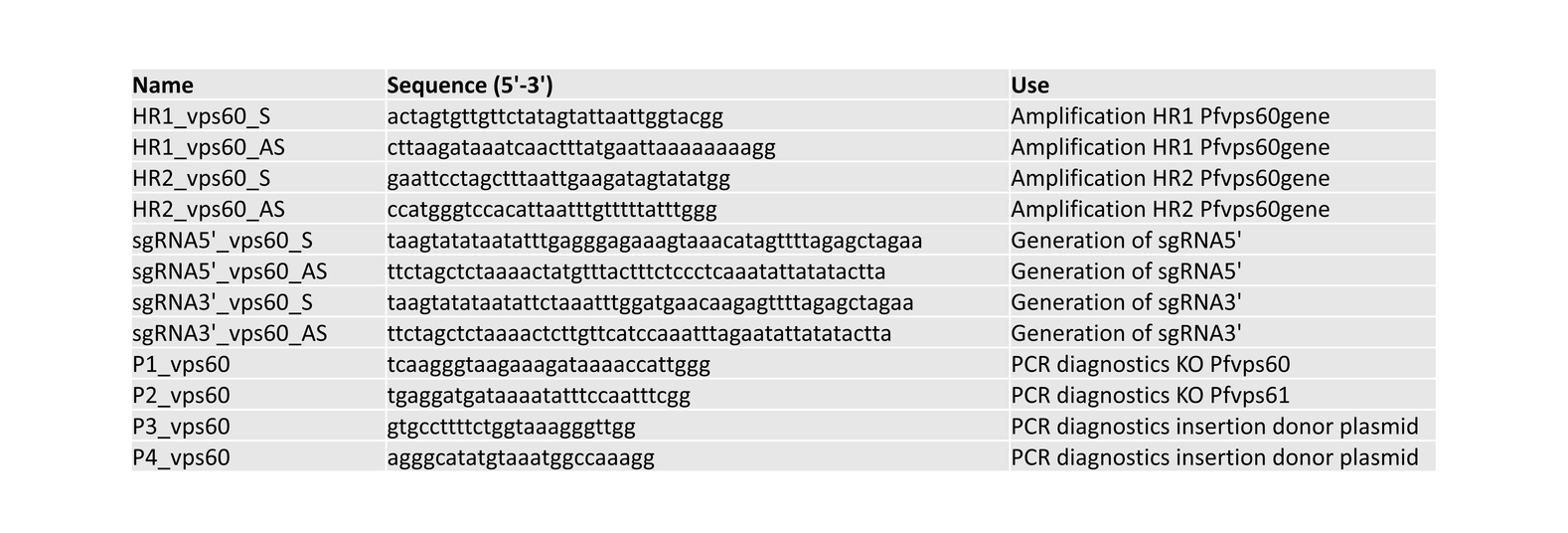

Supplement: S2 Table — (TIF) [file ppat.1009455.s002.tif]

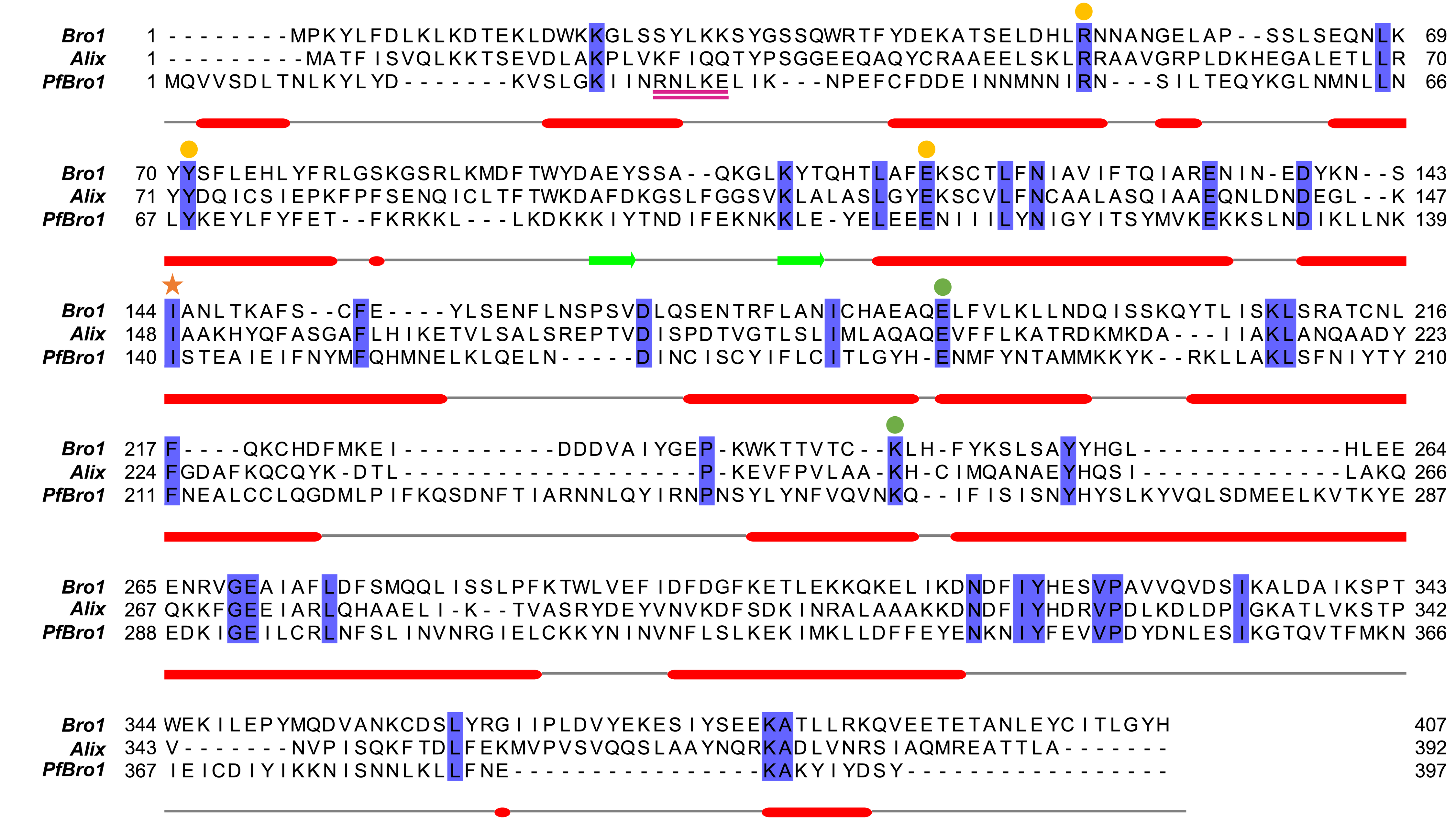

Supplement: S1 Fig — Conserved residues are shadowed in blue. Conserved amino acids present in Bro1-containing proteins are indicated with colored circles, yellow for the polar cluster I and green for the polar cluster II. A key isoleucine involved in Vps32 binding is indicated with a star. The conserved PEXEL motif is double underlined in pink. The secondary structure for PfBro1 is displayed below the sequences, alpha helices represented in red and beta-sheets in green. Sequence alignments were performed with Clustal Omega and edited in Jalview 2. (TIF) [file ppat.1009455.s003.tif]

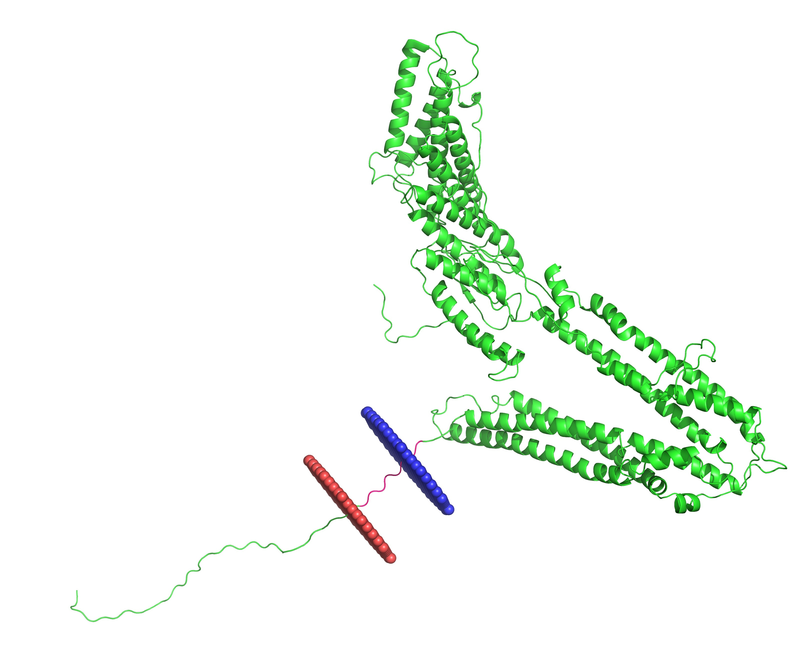

Supplement: S2 Fig — The hydrophobic tail is colored in pink. Outer membrane leaflet is colored in red, inner leaflet in blue. The structure was generated using the Phyre2 server and the OPM database. (TIF) [file ppat.1009455.s004.tif]

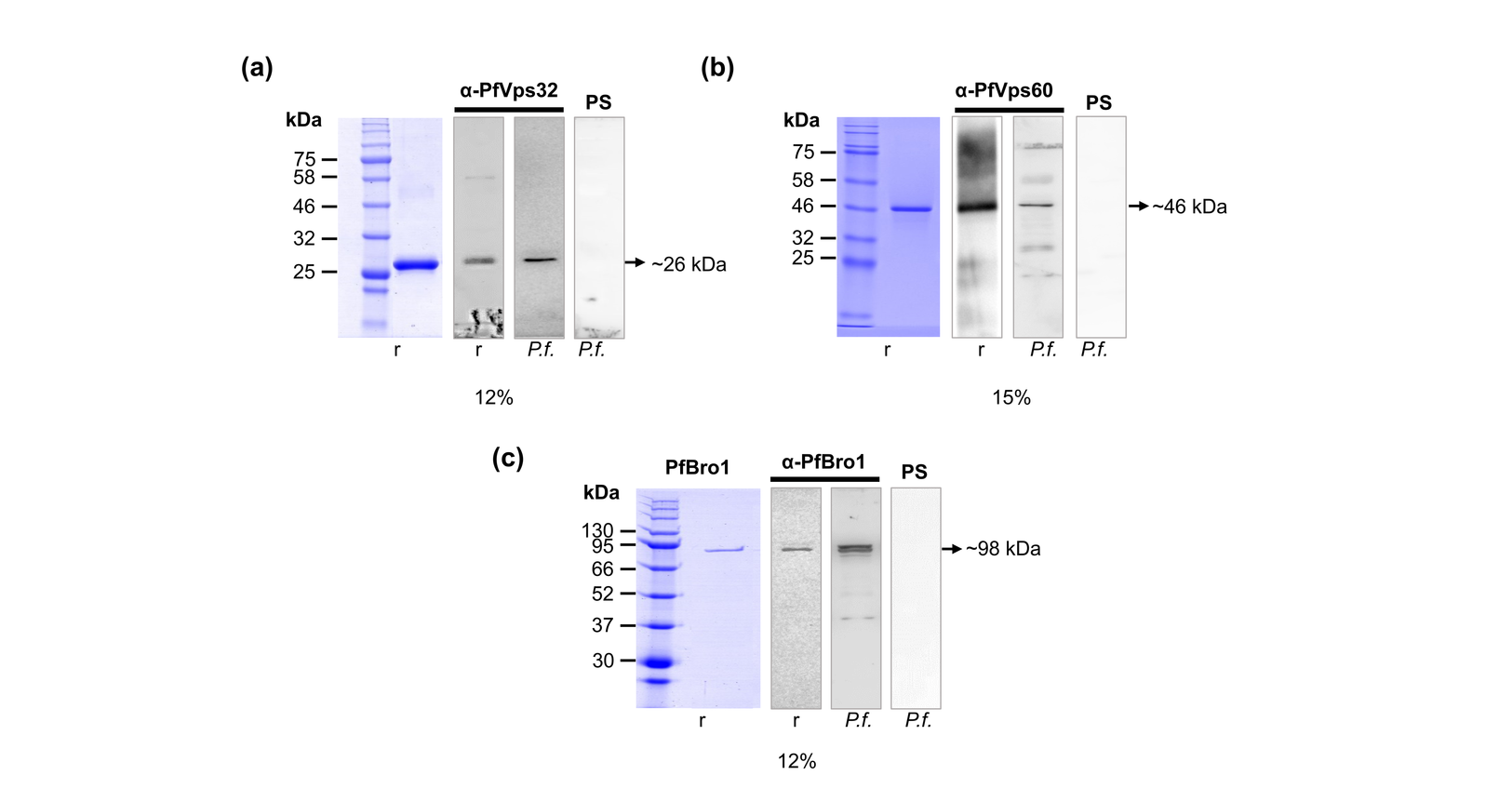

Supplement: S3 Fig — Left panels: SDS-PAGE gels stained with Coomassie blue showing the purified fractions of (a) PfVps32, (b) PfVps60 and (c) PfBro1 that were used for this study. The rest of the panels show Western blot assays of the induced bacterial lysates (r) or P. falciparum-infected RBCs (P.f.) at 30 hours post invasion, using the specific antibodies or preimmune serum (PS) as indicated above the corresponding panels. Arrows indicate the approximate molecular weight. The polyacrylamide percentage is indicated below each gel. (TIF) [file ppat.1009455.s005.tif]

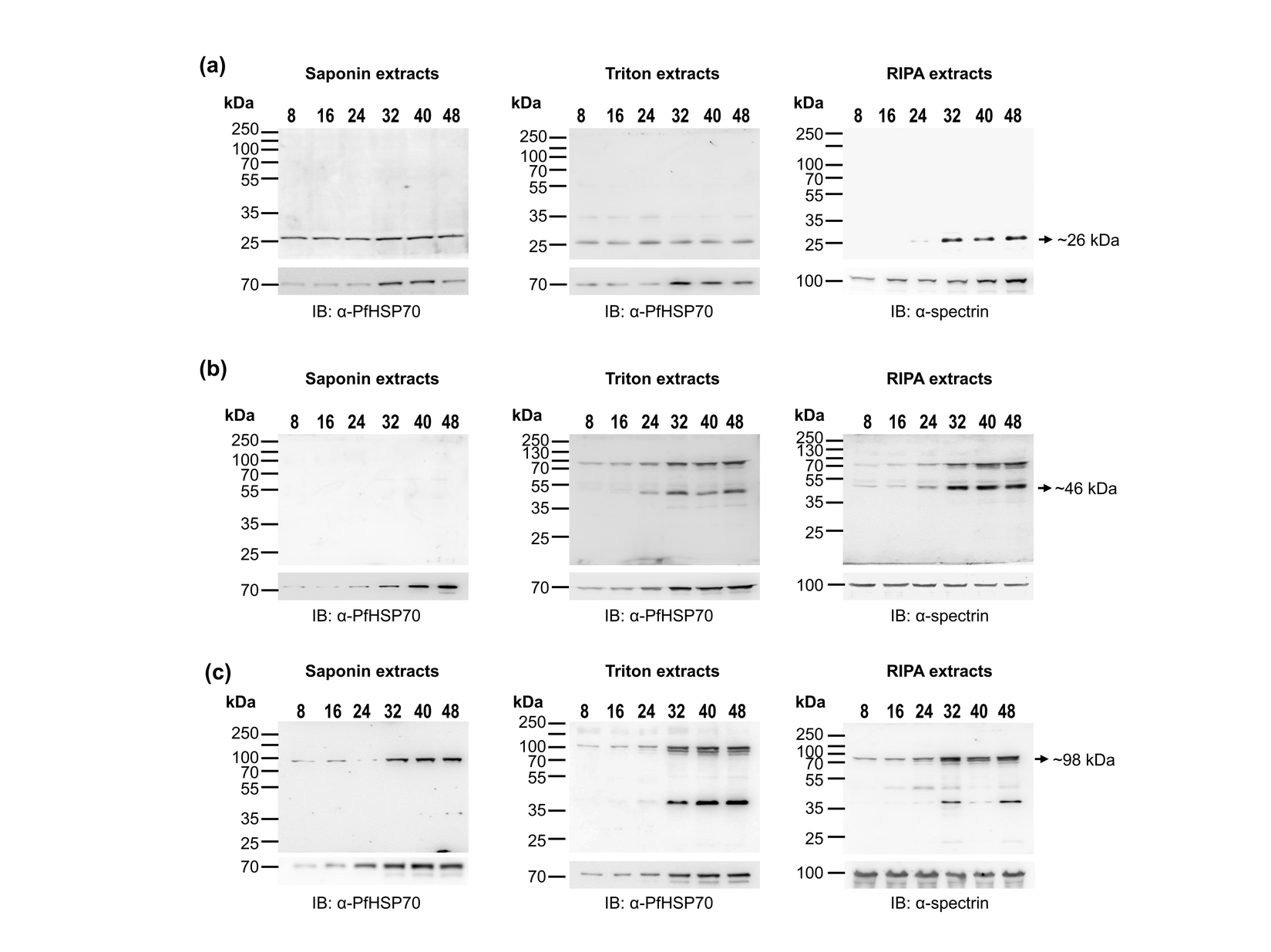

Supplement: S4 Fig — Western blot analysis of P. falciparum in saponin, Triton X-100 or RIPA buffer protein extracts at different hours post invasion (indicated above the upper panels) to monitor protein expression of (a) PfVps32, (b) PfVps60 and (c) PfBro1. Arrows indicate the approximate molecular weight. IB: antibody used for loading control in the lower panels. (TIF) [file ppat.1009455.s006.tif]

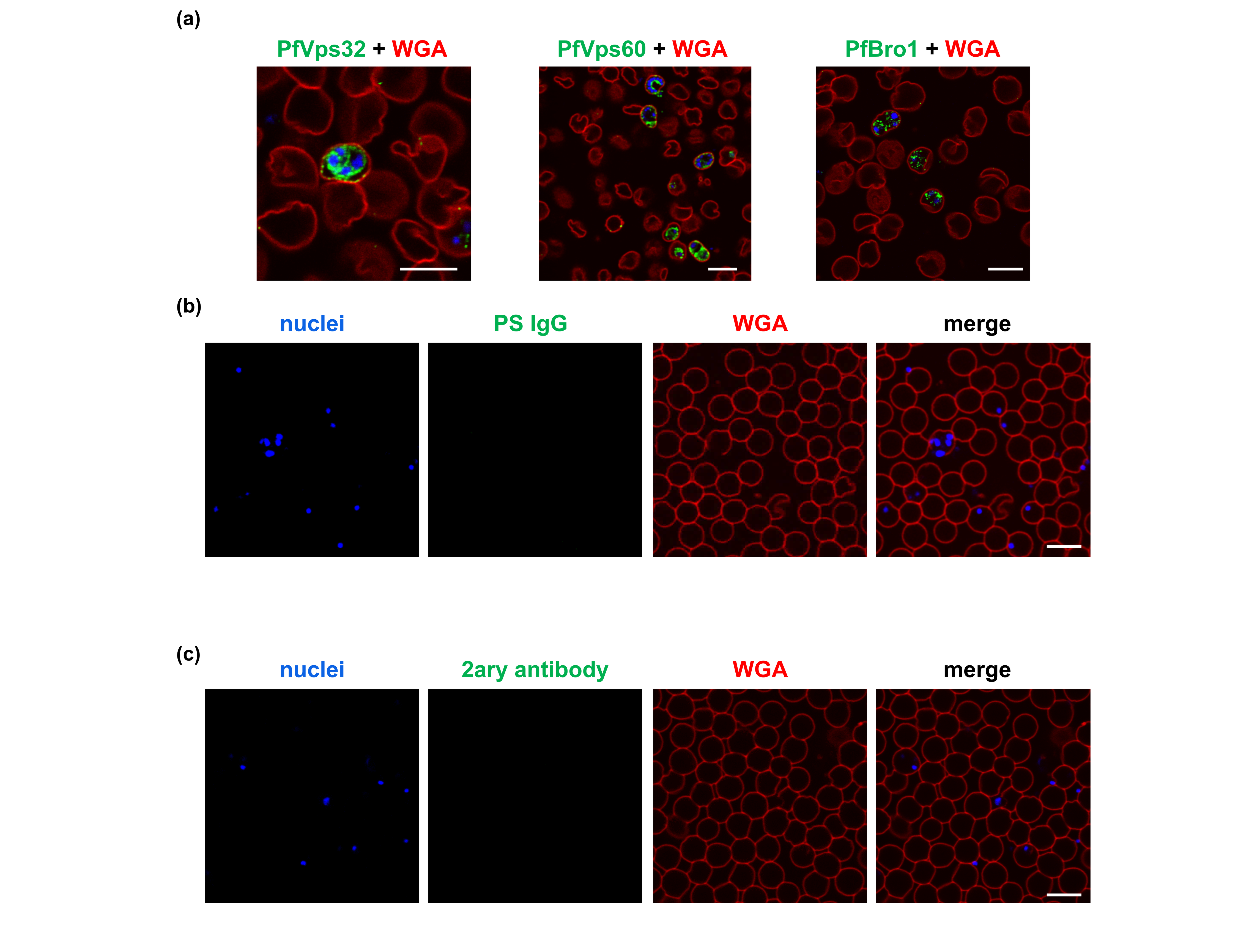

Supplement: S5 Fig — Human erythrocytes were infected with P. falciparum and fixed with 4% PFA. (a) PfVps32, PfVps60 or PfBro1 (green) and WGA (red) were detected by indirect confocal immunofluorescence microscopy using the corresponding specific antibodies. Non-infected red blood cells did not show any antibody recognition. As negative controls, cells were incubated with (b) IgGs purified from preimmune serum (PS) or (c) only the secondary antibody anti-rabbit-Alexa488. Cell nuclei were visualized with Hoechst 33342 (blue). Scale bar: 10 μm. (TIF) [file ppat.1009455.s007.tif]

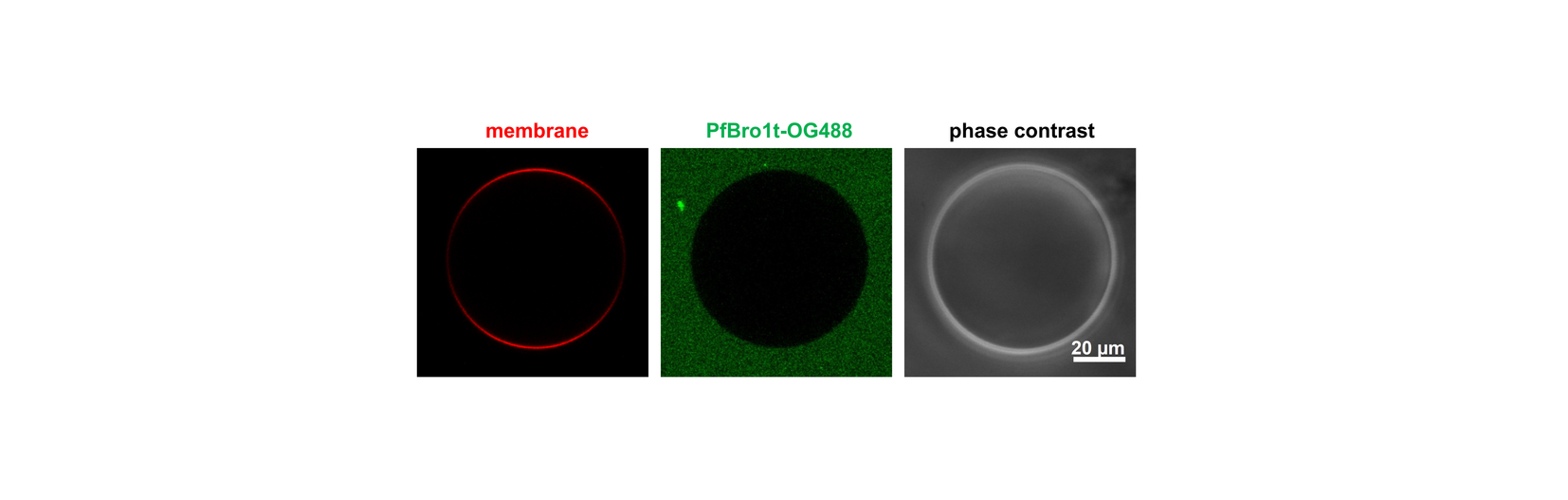

Supplement: S6 Fig — POPC:POPS (80:20) GUVs labeled with DiIC18 were diluted in protein buffer, incubated with 600 nM of PfBro1t in a 1:3 ratio (labeled:unlabeled protein), and visualized by fluorescence confocal microscopy. (TIF) [file ppat.1009455.s008.tif]

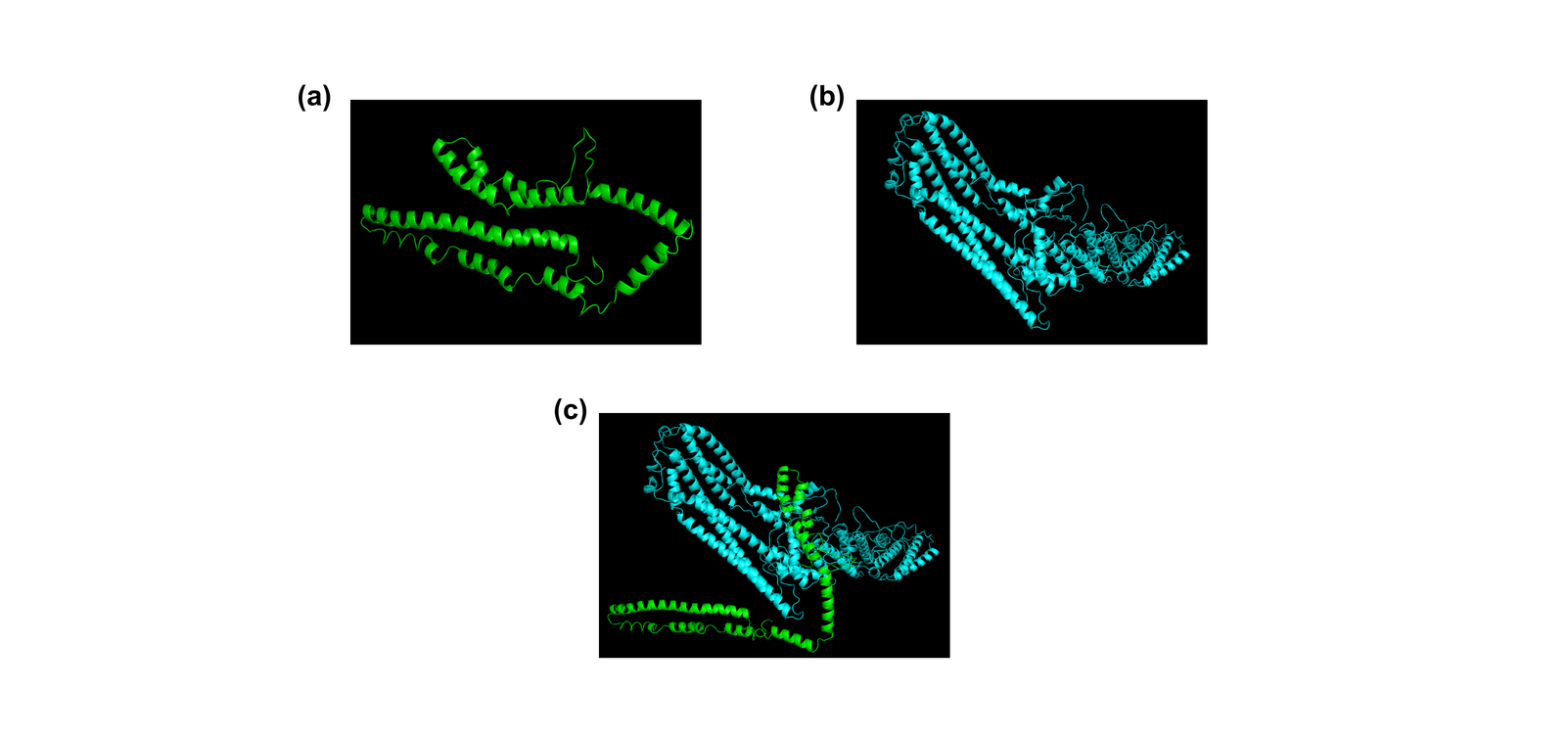

Supplement: S7 Fig — Predicted structure of (a) PfVps60 in its auto-inhibited form and (b) Bro1-domain of PfBro1. (c) Protein docking simulation showing the PfVps60 “opening”. All images were generated using PyMOL. (TIF) [file ppat.1009455.s009.tif]

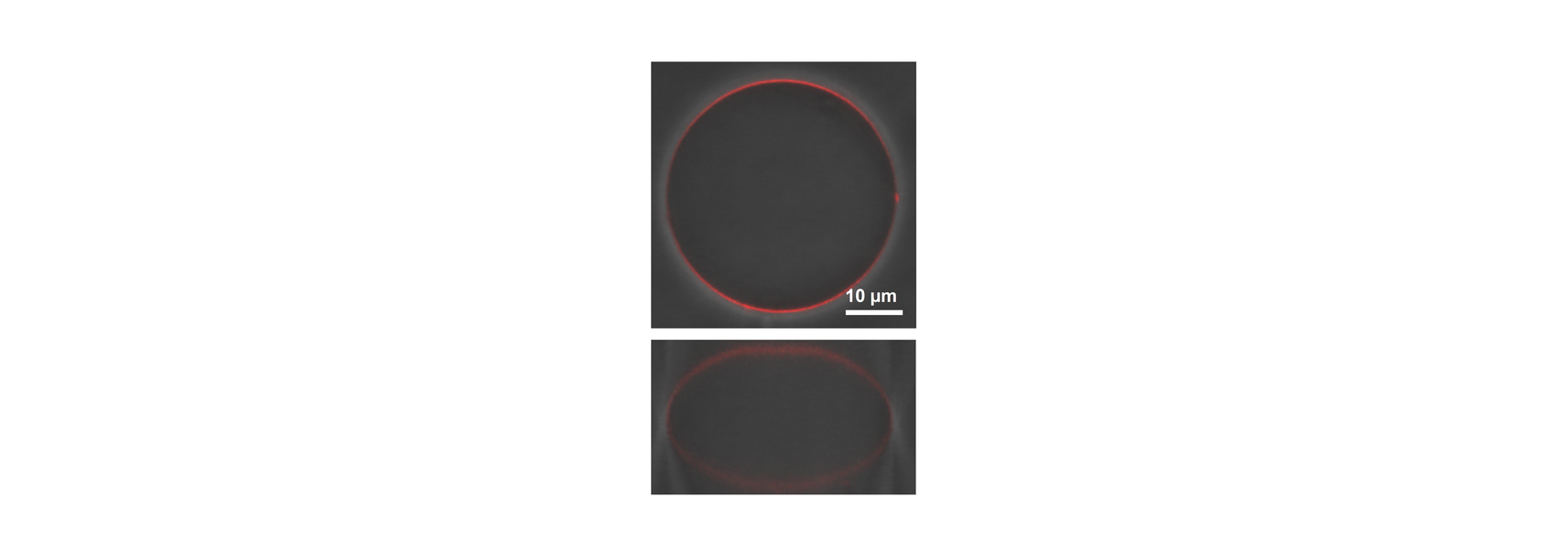

Supplement: S8 Fig — Panels show the top and side view of a typical vesicle selected to perform protein injection. (TIF) [file ppat.1009455.s010.tif]

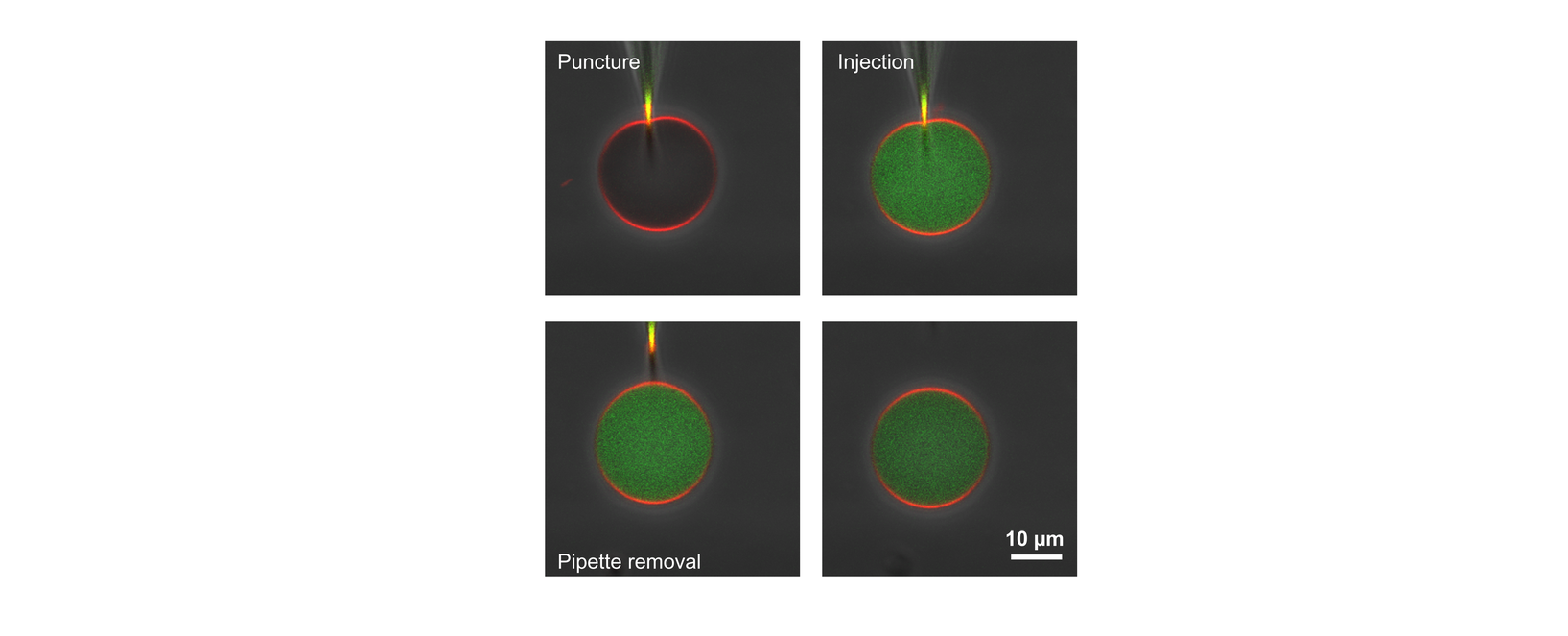

Supplement: S9 Fig — GUVs composed by POPC:POPS:DSPE-biotin (79:20:1) and labeled with DPPE-rhodamine (0.1 mol%) were grown on a PVA substrate using protein buffer, harvested after 10 min and deposited on an avidin-coated coverslip, and injected with PEG-FITC. No alterations were observed up to 5 min after injection. (TIF) [file ppat.1009455.s011.tif]
