# Supplementary material for: The ESCRT-III machinery participates in the production of extracellular vesicles and protein export during Plasmodium falciparum infection
Source: PLoS Pathog. 2021 Apr 2;17(4):e1009455. doi: 10.1371/journal.ppat.1009455 (PMC9159051; doi:10.1371/journal.ppat.1009455)
Supplement: S1 Supporting Materials and Methods — (PDF) [file ppat.1009455.s015.pdf]

The ESCRT-III machinery participates in the production of extracellular vesicles and protein export during *Plasmodium falciparum* infection

Yunuen Avalos-Padilla*[,1,2,3], Vasil N. Georgiev[3], Elena Lantero[1,2], Silvia Pujals[1,4], René Verhoef[5], Livia N. Borgheti-Cardoso[1], Lorenzo Albertazzi[1,6], Rumiana Dimova[3] and Xavier Fernàndez-Busquets*[,1,2]

[1] Institute for Bioengineering of Catalonia (IBEC), The Barcelona Institute of Science and Technology (BIST), Barcelona, Spain

[2] Barcelona Institute for Global Health (ISGlobal, Hospital Clínic-Universitat de Barcelona), Barcelona, Spain

[3] Department of Theory and Bio-Systems, Max Planck Institute of Colloids and Interfaces, Science Park Golm, Potsdam, Germany

[4] Department of Electronics and Biomedical Engineering, Faculty of Physics, Universitat de Barcelona, Barcelona, Spain

[5] Computational Biology Group, Eindhoven University of Technology, Eindhoven, The Netherlands

[6] Department of Biomedical Engineering and the Institute for Complex Molecular Systems, Eindhoven University of Technology, Eindhoven, The Netherlands

*****Corresponding authors: (Y.A.-P.) yavalos@ibecbarcelona.eu,

(X. F.-B.) xfernandez_busquets@ub.edu

Supplemental Materials and Methods

Unless otherwise indicated, reagents were purchased from Sigma-Aldrich (St. Louis, MO, USA).

In silico analysis of PfBro1

The Bro1 protein domain was searched in the *P. falciparum* genome database (http//www.plasmodb.org). Comparison between putative PfBro1 and human and yeast orthologs was determined using the Expert Protein Analysis Systems (ExPASy) Proteomics Server by the NCBI BLAST service program. Sequence alignments were generated using the Clustal Omega program [1] and edited in Jalview [2].

Cloning and expression of PfVps32, PfVps60, PfBro1 and PfBro1t

The proteins PfVps32, PfVps60 and PfBro1 (PlasmoDB accession numbers PF3D7_1243500, PF3D7_1441800 and PF3D7_1224200, respectively) were expressed from a codon-optimized synthetic gene (Genscript, Leiden, The Netherlands). PfVps32 and PfVps60 were inserted into pGEX-6P-1 (GE Healthcare, Freiburg, Germany) as a fusion protein with a Glutathione-S-transferase (GST) tag linked to the N terminus of the proteins via a preScission protease site. PfBro1 was inserted into pET20b(+) as a fusion protein with histidine tag (6×, C-terminal). To generate the truncated *PfBro1t*, the first 397 residues from *PfBro1* were PCR-amplified using plasmid cDNA as template and the specific primers (CC<u>GGATCC</u>GATGCAAGTGGTTAGCGACCTGACCAATCTGAAATAC and CC<u>GTCGAC</u>GTAGCTATCATAGATGTATTTCGCCTTCTCG), which introduced unique *Bam*HI and *Sal*I (underlined) sites in the sense and antisense primers, respectively. The *PfBro1t* gene was cloned into the pJET1.2/blunt plasmid (Thermo Fisher), according to the manufacturer's instructions. Then, *PfBro1t* was subcloned into the pET20b(+) plasmid.

The proteins GST-PfVps32, GST-PfVps60, PfBro1-His(6×) and PfBro1t-His(6×) were expressed in *Escherichia coli* C43 (DE3) for 16 h at 20 °C in Terrific Broth

medium. GST-containing cells were lysed by sonication in lysis buffer (50 mM tris-HCl, pH 8.0, 300 mM NaCl, 1 mM EDTA, 1 mM PEFABLOC SC (Roche, Mannheim, Germany), and 2 mg/ml lysozyme). Cleared lysates were applied to glutathione Sepharose 4B resin (GE healthcare) for at least 5 h at 4 °C under gentle stirring. After this time, the resin was washed 4× with phosphate buffered saline (PBS) and incubated with PreScission protease in cleavage buffer (50 mM tris-HCl, pH 7.5, 150 mM NaCl, 1 mM EDTA, 1 mM dithiothreitol) for 4 h at 4 °C. Following incubation, the GST-free proteins were recovered after centrifugation of the resin. For the His-tagged proteins, cells were lysed by sonication in His-binding buffer (20 mM Na_3PO_4, 500 mM NaCl, 30 mM Imidazole, pH 7.4, 0.2 mg/ml lysozyme, 20 µg/ml DNAse I, 1 mM $MgCl_2$ and 1 mM PEFABLOC SC) as described before. Lysates were applied in HisTrap™ FF crude columns, previously equilibrated with binding buffer. His-tagged proteins were eluted using the His-binding buffer at increasing concentrations of imidazole until reaching 500 mM. The eluted proteins were concentrated and applied to a Superdex 200 10/300 GL column (GE Healthcare) and separated in PBS, pH 7.4. The pooled Superdex 200 fractions were confirmed by sodium dodecyl sulfate-polyacrylamide gel electrophoresis (SDS-PAGE) stained with Coomassie Blue, concentrated and snap frozen in liquid nitrogen in small aliquots until use.

Labelling of recombinant proteins

The purified recombinant proteins were labelled using Oregon Green (OG) 488 (Molecular Probes-Thermo Fisher, Eugene, OR, USA) following the manufacturer's protocol. The labelled and unlabelled proteins were separated by size exclusion chromatography with a Superdex 200 16/600 column (GE healthcare) connected to an Äkta-Purifier FPLC (GE healthcare). The degree of labelling was assessed according to manufacturer's instructions. In all cases, a 1:3 ratio of labelled:unlabelled proteins was used to maintain protein activity.

Generation and labelling of polyclonal antibodies

PfBro1 (400 µg), PfVps32 and PfVps60 (both 413 µg) were emulsified separately in complete Freund's adjuvant (1:1) and inoculated into *New Zealand* male rabbits. Three more doses of 200 µg of each protein emulsified in incomplete Freund's

adjuvant were injected at 21-day intervals and animals were bled to obtain α-PfVps32, α-PfVps60 and α-PfBro1 polyclonal antibodies. Polyclonal IgGs were purified using protein A sepharose affinity chromatography. Pre-immune serum was obtained before immunization in all cases for control experiments. Experimental procedures were approved by the Ethical Committee of Animal Experimentation of the Universitat de Barcelona with registration number 10094, and performed in accordance with Spanish legislation and also with the European Union directives (2010 / 63 / EU).

The labelling of the antibodies for the co-localization experiments was performed using either Alexa Fluor 647 NHS ester or Alexa Fluor 488 NHS ester (Thermo Scientific) according to the manufacturer's instructions. Briefly, 0.1 ml of 1 M $NaHCO_3$ were added to 5 mg of antibody solution, and the mixture was incubated with the different fluorophores overnight at 4 °C. The free dye was removed using a desalting PD-10 column and PBS.

Subcellular protein extraction

For the analysis of PfVps32, PfVps60 and PfBro1 throughout the whole intraerythrocytic cycle of *P. falciparum*, tightly synchronized cultures (8, 16, 24, 32, 40 and 48 hpi) were obtained as described in the main manuscript. Then, samples from each time point underwent differential detergent fractionation as previously described [3]. Briefly, *Plasmodium*-infected RBC pellets were washed with PBS-complete buffer (PBS supplemented with 1× cOmplete protease inhibitor cocktail, Roche). Then, pellets were suspended in 6× volumes of 0.15% saponin for 10 min at 4 °C and centrifuged (10,000× g, 15 min, 4 °C); the resulting supernatant contained cytosolic soluble proteins. The remaining pellet was washed with PBS-complete and incubated in 1% Triton X-100 for 30 min at 4 °C. Again, samples were centrifuged (20,000× g, 30 min, 4 °C) and the new supernatant containing membrane and organelle proteins was recovered. Finally, Triton-insoluble pellets were incubated for 30 min with 100 µl of RIPA buffer (40 mM tris-HCl, pH 7.4, 150 mM NaCl, 2 mM EDTA, 10% glycerol, 1% Triton X-100, 0.5% sodium deoxycholate, 0.2% SDS and 1× cOmplete protease inhibitor cocktail) under continuous and vigorous

shaking at 4 °C. Extracts were sonicated for 2 s at 40% amplitude and incubated on ice for 15 min. Finally, extracts were centrifuged at 20,000× g to eliminate undissolved molecular debris. The resulting supernatant contained nuclear and detergent-resistant cytoskeletal/matrix proteins.

Dot blots and Western blots

Protein extracts from pRBCs and RBCs obtained by detergent fractionation were deposited onto nitrocellulose membranes in concentrations ranging from 20 µg for the saponin fraction to 2 µg for the Triton X-100 and RIPA buffer fractions, and dots were air-dried. Then, membranes were blocked overnight with 5% skim milk dissolved in Tris Buffered Saline (TBS: 50 mM tris-HCl, pH 7.6, 150 mM NaCl) containing 0.5% Tween 20. Membranes were probed with either rabbit α-PfVps32 (1:10,000), rabbit α-PfVps60 (1:12,000), rabbit α-PfBro1 (1:8,000), rabbit α-PfHSP70 (StressMarq, 1:10,000), rabbit α-Glycophorin A (1:5,000) or mouse α-spectrin alpha/beta (1:10,000) antibodies for 3 h at room temperature (RT). After this time, membranes were washed and incubated for 1 h at RT with α-rabbit or α-mouse HRP-labelled secondary antibodies (Abcam, 1:10,000) and developed with ECL Prime Western blotting detection reagent (GE Healthcare). Dot blot analysis was performed using a LAS-4000 image reader (GE Healthcare). For Western blots, protein extracts (20 µg) of each time point were separated in a 12% SDS-PAGE, transferred to a polyvinylidene difluoride membrane (BioRad, Hercules, CA, USA) and blocked with 5% skim milk dissolved in TBS containing 0.5% Tween 20. Membranes were probed with the different antibodies and processed as for dot blot assays.

Light microscopy and image processing

For indirect immunofluorescence detection of PfVps32, PfVps60 and PfBro1, thin blood smears of synchronized cultures at different hpi were air-dried and fixed with a 9:1 mixture of acetone:methanol for 2 min at RT. Slides were thoroughly washed with PBS and incubated for 1 h at RT with the primary antibodies α-PfBro1, α-PfVps32 and α-PfVps60 diluted 1:200 in PBS supplemented with 0.75% bovine

serum albumin (BSA). After washing with PBS, Alexa Fluor 488-labelled secondary α-rabbit IgG antibodies were used at 1:500 dilution. Lectins present on the RBC surface were stained using WGA-rhodamine (Invitrogen, 1:500), and nuclei were counterstained with Hoechst 33342 (1:5,000). All preparations were preserved using ProLong gold antifade reagent (Invitrogen) and images were collected with a Leica TCS SP5 confocal microscope (Mannheim, Germany) using a 63× oil immersion objective. All immunofluorescence assays were repeated three times. To quantify Manders' overlap coefficient [4], images were analyzed using the Just Another Colocalization Plugin (JACoP) [5] in the Fiji software [6].

Inmunoprecipitation

P. falciparum-infected RBCs at 30 hpi and 3.5% parasitemia were washed twice with PBS-complete buffer and the pellet was treated with 0.07% saponin for 10 min at 4 °C to remove excess of hemoglobin. Then, the cell pellet was washed twice with PBS-complete buffer and proteins were extracted with RIPA buffer as described previously. In parallel, 200 µl of recombinant protein G-agarose beads (Invitrogen) were incubated with 100 µg of purified rabbit α-PfBro1 IgG antibodies or preimmune serum for 2 h at 4 °C, with gentle stirring. Then, beads were washed with 0.5% BSA in PBS, followed by three more washes with PBS under gentle stirring, and centrifuged at 11,000× g for 2 min. Afterwards, pRBC lysates (1 mg) were pre-cleared with 200 µl of beads (blocked previously with 2% BSA) and incubated for 2 h at 4 °C under gentle stirring. Samples were centrifuged at 11,000× g to obtain the supernatant that was added to Protein-G beads previously incubated with the purified IgGs and incubated overnight at 4 °C. Finally, beads were recovered by centrifugation and washed four times with PBS. Samples were mixed with 4× Laemmli buffer (40% glycerol, 240 mM tris-HCl, pH 6.8, 8% SDS, 0.04% Bromophenol Blue and 5% β-mercaptoethanol) and processed for electrophoresis and Western blot as described previously.

Simulations

All simulations were executed on the 'hot' computer cluster of the Max Planck Institute of Colloids and Interfaces. The Rosetta software suite version 3.6 was used

for structure refining and protein docking simulations. Visualizations of protein structures were produced using pyMOL with custom Python scripts. For all data analyses and curvature calculations custom Python scripts were written.

Structure refining

Rough structure predictions of PfBro1 and PfVps32 were acquired from the Phyre2 server [7] (http://www.sbg.bio.ic.ac.uk/phyre2/html/page.cgi?id=index) and subjected to structure refining to prepare both proteins for docking and adapt them to the Rosetta force field. A total of 11,652 PfBro1 and 27,520 PfVps32 structures were generated with a standard FastRelax protocol. Given the rigidity and size of the PfBro1 protein (819 residues), a further refinement in a backrub simulation was performed where 200 structures were generated, of which the best 20 structures from the relax ensemble were chosen based on their total score.

Protein docking

Potential orientations for protein docking of PfBro1 and PfVps32 were previously acquired using the ClusPro web server (https://cluspro.org) [8], omitting the need for global docking simulations. As input we used two PfVps32 structures in 'open' state and a good scoring PfBro1 structure. For docking simulations these structures were aligned with a prominent prediction from ClusPro using pyMOL. To remove any steric hindrances that could be present due to our manual construction of the protein-protein system, a quick restrained relaxation was performed with 96 structures each, the best of which were chosen for the following docking simulations. After performing a prepacking protocol (also with the 96 best scoring structures), local docking simulations were performed generating 6,144 structures for each system. Out of these, the 1,000 lowest scoring structures were filtered using a low-pass filter on an interface score of minus 5 REU. The ca. 20 structures per system that remained after filtering were then further refined by performing additional backrubbing simulations, once again generating 200 structures for each filtered structure.

References

1.Sievers F, Higgins DG. Clustal Omega for making accurate alignments of many protein sequences. Protein Sci. 2018;27(1):135-45. doi: 10.1002/pro.3290. PubMed PMID: WOS:000418254300014.

2.Waterhouse AM, Procter JB, Martin DMA, Clamp M, Barton GJ. Jalview Version 2-a multiple sequence alignment editor and analysis workbench. Bioinformatics. 2009;25(9):1189-91. doi: 10.1093/bioinformatics/btp033. PubMed PMID: WOS:000265523300016.
3.Ramsby M, Makowski G. Differential detergent fractionation of eukaryotic cells. Cold Spring Harb Protoc. 2011;2011(3):prot5592. Epub 2011/03/03. doi: 10.1101/pdb.prot5592. PubMed PMID: 21363956.
4.Manders EMM, Verbeek FJ, Aten JA. Measurement of co-localization of objects in dual-color confocal images. Journal of Microscopy. 1993;169:375-82. doi: https://doi.org/10.1111/j.1365-2818.1993.tb03313.x.
5.Bolte S, Cordelieres FP. A guided tour into subcellular colocalization analysis in light microscopy. Journal of Microscopy-Oxford. 2006;224:213-32. doi: DOI 10.1111/j.1365-2818.2006.01706.x. PubMed PMID: WOS:000242993500001.
6.Schindelin J, Arganda-Carreras I, Frise E, Kaynig V, Longair M, Pietzsch T, et al. Fiji: an open-source platform for biological-image analysis. Nature Methods. 2012;9(7):676-82. doi: 10.1038/Nmeth.2019. PubMed PMID: WOS:000305942200021.
7.Kelley LA, Mezulis S, Yates CM, Wass MN, Sternberg MJE. The Phyre2 web portal for protein modeling, prediction and analysis. Nature Protocols. 2015;10(6):845-58. doi: 10.1038/nprot.2015.053. PubMed PMID: WOS:000355245200002.
8.Kozakov D, Hall DR, Xia B, Porter KA, Padhorny D, Yueh C, et al. The ClusPro web server for protein-protein docking. Nature Protocols. 2017;12(2):255-78. doi: 10.1038/nprot.2016.169. PubMed PMID: WOS:000394187000005.
